# A plant-based diet supplemented with *Hermetia illucens* alone or in combination with poultry by-product meal: one step closer to sustainable aquafeeds for European seabass

**DOI:** 10.1186/s40104-022-00725-z

**Published:** 2022-07-11

**Authors:** Ivana Lepen Pleić, Ivana Bušelić, Maria Messina, Jerko Hrabar, Luka Žuvić, Igor Talijančić, Iva Žužul, Tina Pavelin, Ivana Anđelić, Jelka Pleadin, Jasna Puizina, Leon Grubišić, Emilio Tibaldi, Tanja Šegvić-Bubić

**Affiliations:** 1 Laboratory of Aquaculture, Institute of Oceanography and Fisheries, Šetalište I. Meštrovića 63, 21000 Split, Croatia; 2 Department of Agriculture, Food, Environmental and Animal Sciences, University of Udine, Udine, Italy; 3 Department of Chemistry, Faculty of Science, University of Split, Ruđera Boškovića 33, 21000 Split, Croatia; 4 Croatian Veterinary Institute, Laboratory for Analytical Chemistry, Savska Cesta 143, 10000, Zagreb, Croatia; 5 Department of Biology, Faculty of Science, University of Split, Ruđera Boškovića 33, 21000 Split, Croatia

**Keywords:** Avian by-product meal, Circular economy, *Dicentrarchus labrax*, Fitness, Growth, Insect

## Abstract

**Background:**

Increasing demand for high-value fish species and pressure on forage fish is challenging aquaculture to ensure sustainable growth by replacing protein sources in aquafeeds with plant and terrestrial animal proteins, without compromising the economic value and quality of the final fish product. In the present study, the effects of a plant protein-based diet (CV), two plant-based diets in which graded amounts of plan protein mixtures were replaced with *Hermetia illucens* meal alone (VH10) or in combination with poultry by-product meal (PBM) (VH10P30), a fishmeal (FM) diet (CF) and an FM diet supplemented with *H. illucens* (FH10) on growth performance, gut health and homeostasis of farmed subadult European seabass were tested and compared.

**Results:**

Fish fed the VH10 and VH10P30 diets showed the highest specific growth rates and lowest feed conversion ratios among the tested groups. Expectedly, the best preservation of PI morphology was observed in fish fed the CF or FH10 diets, while fish fed the CV diet exhibited significant degenerative changes in the proximal and distal intestines. However, PBM supplementation mitigated these effects and significantly improved all gut morphometric parameters in the VH10P30 group. Partial substitution of the plant mixture with insect meal alone or PBM also induced most BBM genes and activated BBM enzymes, suggesting a beneficial effect on intestinal digestive/absorption functions. Regarding intestinal microbiota, fish fed diets containing *H. illucens* meal (FH10, VH10, VH10P30) had the highest richness of bacterial communities and abundance of beneficial genera such as *Lactobacillus* and *Bacillus*. On the other hand, fish fed CV had the highest microbial diversity but lost a significant component of fish intestinal microbiota, the phylum Bacteroidetes. Finally, skin pigmentation most similar to that of farmed or even wild seabass was also observed in the fish groups fed CF, FH10 or VH10P30.

**Conclusion:**

Plant-based diets supplemented with PBM and *H. illucens* pupae meal have great potential as alternative diets for European seabass, without affecting growth performance, gut homeostasis, or overall fitness. This also highlights the importance of animal proteins in diets of European seabass, as the addition of a small amount of these alternative animal protein sources significantly improved all measured parameters.

**Supplementary Information:**

The online version contains supplementary material available at 10.1186/s40104-022-00725-z.

## Introduction

Over the last three decades, aquaculture has been considered the fastest-growing global food sector and source of animal protein for human consumption. About 70% of aquaculture production depends on providing aquatic animals with high quality protein-rich aquafeeds [1]. For resource-intensive carnivorous species such as European seabass (*Dicentrarchus labrax* L.) and gilthead seabream (*Sparus aurata* L.), aquafeeds have historically relied more on fish meal (FM) and fish oil (FO), as nearly optimal protein and lipid sources from the nutritional standpoint, and less on by-products. However, the growing pressure on small pelagic fish, i.e., forage fish used for crushing into FM and extracting FO, has contributed to a progressive decline in these fish stocks [1, 2], leading to a significant increase in the market price of FM and FO. Given the increased demand for high-value fish species, aquaculture faces the challenge of ensuring sustainable growth by replacing fish protein sources with plant and terrestrial animal proteins, without compromising the economic value and quality of the final fish product [3–5]. These alternative sources should meet the nutritional needs of fish while being economically affordable and environmentally sustainable.

Farmed European seabass is considered one of the most economically important fish species in the European Union, with aquaculture accounting for 96% of total fish production [6]. Over the years, numerous plant-based feeds obtained from soybean, maize, wheat, rapeseed, and pea have been investigated as alternative sources of protein and oil, leading to partial replacement of FM without negative effects on seabass survival, growth performance, and gut health [7–9]. However, the use of terrestrial plant proteins is often associated with several disadvantages, such as decreased palatability and digestibility [10, 11], inadequate fatty acid profile [12] and the presence of anti-nutritional factors [13]. In addition, land-based crop production is associated with deforestation, high water consumption, and intensive use of fertilisers and pesticides [14]. Other alternative sources such as processed animal proteins (PAPs) from non-ruminants, reintroduced as legal aquafeed ingredients in 2013 (Commission Regulation (EU) No 56/2013), proved to be a suitable alternative to FM protein because of their high protein content and quality, mineral content, and suitable palatability [15, 16]. Together with fisheries trimmings and by-products as a protein source, their use supports industrial sustainability and circular economy principles [17]. Nevertheless, the complete replacement of conventional protein sources with poultry by-product meal (PBM) as the most common source in the seabass diet is not yet achievable due to the lower availability of essential amino acids such as methionine and lysine [18] and due to the presence of poultry fat, which is rich in monounsaturated fatty acids and n-6 PUFA but low in essential fatty acids such as n-3 LC-PUFA, EPA, and DHA [16]. This is often quoted as the reason for reduced growth and significantly higher feed conversion in certain species when fed diets high in PBM [16]. As a result, several aquafeed ingredients such as macroalgae, probiotic bacteria, yeasts and insects have been developed and intensively evaluated [19–23]. Insect meal has attracted the interest of animal nutritionists due to their moderate to high protein content, ranging from 35% to 60% dry weight or 10–25% fresh weight. This is in the upper range, exceeding even meat and chicken eggs [24], while being relatively easily digestible [25, 26]. Since the EU (Commission Regulation (EU) 2017/893) [27] permitted the use of larval insect meal (e.g., black soldier fly *Hermetia illucens* and yellow mealworm *Tenebrio molitor*) for aquafeed in 2017, several studies on *H. illucens* and *T. molitor* have shown that there are no side effects on the growth performance of European seabass at inclusion rates of 20–30% [28–30]. However, the fatty acid content of insect meal can vary depending on the composition of the substrate and developmental stage of the insect, though it mostly depends on insect species, e.g., *H. illucens* is rich in n-3 polyunsaturated fatty acids, whereas *T. molitor* is abundant in n-6 polyunsaturated fatty acids [31]. Consequently, the choice of insect meal used in the fish diet can affect the whole-body fatty acid composition of fish [28]. When compared to FO, terrestrial insects contain higher levels of n-6 polyunsaturated fatty acids, but unsubstantial amounts of EPA and DHA. The latter is a limiting factor when using terrestrial insects in marine fish, due to the limited capabilities of fish to synthetise it [32]. Furthermore, the importance of the feeding substrate for insects must be emphasised, since the nutritional values of insects and the possible accumulation of contaminants is directly dependent on the substrate. Namely, *H. illucens* larvae can accumulate heavy metals if present in their feeding substrates [33, 34] which further stresses the importance of rearing substrate selection and its quality. Finally, although insects can grow on various substrates with high productivity and a low ecological footprint, their production is currently not price-competitive, and their admixture in seabass diets increases feeding costs [35]. However, the growing market of insect meals is creating reasonable expectations for the gradual alignment of their prices to those of conventional protein-rich feeds.

In light of the above, novel aquafeed formulations containing sustainable alternatives should strive to meet both environmental and economic sustainability criteria by reducing environmental impacts while remaining commercially viable [17, 36]. To date, the effects of the partial replacement of conventional feed protein sources (FM and protein-rich plant derivatives) with the combination of PBM and insect meal on growth performance and gut health of seabass are not well known. Recent studies have shown that both PBM and *H. illucens* meal can be successfully used to replace plant-derived ingredients in FM-free diets for gilthead seabream [37], while in juvenile seabass, replacing 15% protein of a plant-based formulation with insect or yeast proteins resulted in significantly higher growth performance and feed intake of fish compared to the full plant-based formulation, with the resulting feed conversion similar to a commercial diet [38].

The present study tested and compared the effects of a plant protein-based diet, in which graded levels of the plant protein mixture were replaced with *H. illucens* meal alone or in combination with poultry by-product meal (PBM), and an FM-based diet supplemented exclusively with *H. illucens* on growth performance, gut health and homeostasis in subadult seabass. Applying a multidisciplinary approach, growth performance, muscle tissue composition, skin coloration, intestinal morpho-physiology, digestive enzyme activities, gut microbiota, and feed costs were analysed to search for deeper insight into the potential of new feed formulations as a sustainable alternative feed for farmed European seabass.

## Methods

### Growth trial

#### Diet formulation

Five experimental diets were formulated to be iso-proteic (45%), iso-lipidic (20%), and grossly iso-energetic (20.3 MJ/kg) and to meet all known nutritional requirements of subadult European seabass [39]. Different arrays of protein and lipid sources from fish, plant, insect meal (*Hermetia illucens*), and poultry by-products were used (Table 1). A control diet high in plant-derived ingredients (CV) was formulated to supply 85% and 66% plant protein and lipids. In the following two diets, given proportions of crude protein from plant sources of the diet CV were replaced by 10% crude protein from partially defatted *H. illucens* pupae meal, solely (VH10) or in combination with 30% crude protein from PBM (VH10P30) (Additional file 1: Table S1). In these latter diets, the ratio between plant and fish lipids was the same as in the CV diet. A second control diet, high in fish-derived ingredients (CF), was formulated to contain 85% and 66% fish protein and lipid, respectively. Finally, a fifth diet (FH10) was obtained from the latter preparation by including *H. illucens* pupae meal to replace 10% of the crude protein from plant sources, while maintaining the same ratio of fish to plant lipids as in the CF diet. All diets were manufactured by extrusion at SPAROS Lda (Portugal) using commercial feed ingredients. Feed formula costs (prior to Covid-Sars2) were estimated as: CV–0.967 €/kg; VH10–1.059 €/kg; VH10P30–0.869 €/kg; CF–1.091 €/kg; FH10–1.177 €/kg. Two commonly used indicators were calculated: Fish-In-Fish-Out Ratio (FIFO), which estimates the amount of marine ingredients in the feed, and relative Economic Conversion Ratio (ECR), which measures the cost-effectiveness of fish feed. For more details, see Additional file 1: Supplementary methods.

**Table 1:** Ingredient composition (g/100 g) and proximate (% of fed) of the test diets used for European seabass subadults

Ingredient composition	CV	VH10	VH10P30	FH10	CF
Fish meal^1^	–	–	–	16	16
Fish meal^2^	4	4	4	45	45
Vegetable-protein mix 1^3^	41	33.6	13.1	–	6
Vegetable-protein mix 2^4^	20	20	20		
*Hermentia illucens* meal^5^	–	8	8	8	–
PBM^6^	–	–	20.2	–	–
Feeding stimulants^7^	5.5	5.5	5.5	–	–
Wheat meal*	1	2.6	6.7	8.2	5.7
Whole peas*	6.7	6	5	8	11
Fish oil^8^	6	5.6	6	7.5	8
Vegetable oil mix^9^	11.5	10.4	7.7	5.4	6.4
Vitamin and mineral premix^10^	0.3	0.3	0.3	0.3	0.3
Choline HCl	0.1	0.1	0.1	0.1	0.1
Sodium phosphate (NaH_2_PO4)	1.6	1.6	1.6	–	–
L-Lysine^11^	0.4	0.4	–	–	–
DL-Methionine^12^	0.4	0.4	0.3	–	–
Celite	1.5	1.5	1.5	1.5	1.5
**Proximate composition**					
Crude protein (N × 6.25)	45.2	45.3	45.1	45.5	45.4
Crude lipid	20.1	20.1	20.2	20.3	20.2
Starch^13^	6.2	6.5	7.6	8.4	8.6
Carbohydrate^14^	23.2	21.2	19.5	17.8	17.8
Moisture	4.4	5.7	5.7	4.3	4.7
Ash	7.1	7.7	9.5	12.1	11.9
Gross energy, MJ/kg DM	23.0	23.1	22.9	22.8	22.8

^1^Fishmeal Super Prime - Pesquera Diamante Peru (66.3% crude protein (CP), 11.5% crude fat (CF)). ^2^Fishmeal by-product Conresa 60, Conserveros Reunidos S.A. Spain (61.2% CP, 8.4% CF). ^3^Vegetable-protein source mixture 1 (% composition): soy protein concentrate-Soycomil, 49; wheat gluten, 29; corn gluten, 22. ^4^Vegetable-protein source mixture 2 (% composition): dehulled solvent extracted soybean meal, 65; defatted rapeseed meal, 35. ^5^ProteinX™, Protix, Dongen, The Netherlands (CP, 55.4%; CF, 20.8% as fed). ^6^Poultry by-product meal from Azienda Agricola Tre Valli; Verona, Italy (CP, 65.6%; CF, 14.8% as fed). ^7^Feeding stimulants (% composition): fish protein concentrate CPSP90- Sopropeche, France (82.6% CP), 64; Squid meal (80.3% CP), 36. ^8^Fish oil: from pleagic forage fish, Sopropêche, France. ^9^Vegetable oil mix (% composition): rapeseed oil, 56; linseed oil, 26; palm oil, 18. ^10^Vitamin and mineral supplement (per kg of premix): Vit. A, 2,000,000 IU; Vit D_3_, 200,000 IU; Vit. E 30,000 mg; Vit. K_3_, 2500 mg; Vit.B_1_, 3000 mg; Vit. B_2_, 3000 mg: Vit B_3_, 20,000 mg; Vit. B_5_, 10,000 mg; Vit B_6_, 2000 mg, Vit. B_9_, 1500 mg; Vit. B_12_, 10 mg, Biotin, 300 mg; Stay C®, 90,000 mg; Inositol, 200,000 mg; Cu, 900 mg; Fe, 6000 mg; I, 400 mg; Se, 40 mg; Zn, 7500 mg. ^11^L-lysine, 99%; Ajinomoto EUROLYSINE S.A.S; France. ^12^DL-Methionine: 99%; EVONIK Nutrition & Care GmbH; Germany. ^13^Calculated from the starch content of single ingredients. ^14^ Calculated by difference. *The ingredients were obtained from local providers by Sparos Lda

#### Experimental conditions and sample collection

The feeding trial was carried out in the outdoor water open-circuit system of the Laboratory of Aquaculture, Institute of Oceanography and Fisheries (Split, Croatia). The experiment used 550 subadult European seabass obtained from a local farm (Sardina d.o.o., Brač Island, Croatia). Fish were adapted over two weeks to culture conditions while being fed commercial feed (OptibassL-2, Skretting, Spain; 48.5% proteins, 16% lipids, 3.7% fibre, 6.4% ash) before the start of the feeding trial. After acclimation, fish were measured by total length, weighed, and randomly distributed among 10 concrete 600-l tanks (55 fish per tank), for two replicates for each of the five dietary treatments. Initial mean body weight (± SEM) of 149 ± 1.04 g was not significantly different among tanks (F = 0.71, df = 9, *P* = 0.7). Each diet was randomly assigned to two tanks. The trial started in July 2019 and lasted 21 weeks with monthly weighing and measuring. Fish were hand-fed with the experimental diets in two daily meals (8:00 and 16:00 h) until the first pellet was refused. Feed intake and water parameters were measured daily. Feeding was withheld 24 h before each morphometric measurement and before the last sampling at the end of the trial. Zoo-technical details of the trial are provided in Additional file 1: Fig. S1. At the end of the trial, fish were anesthetised with 50 mg/L MS-222 [40, 41] (Sigma Aldrich, Saint-Louis, MO, USA) for final weighing and digital imaging, while fish required for biological samples were sacrificed with an overdose of MS-222 (500 mg/L) [40, 41] to collect different tissues for analysis. From each tank, the gastrointestinal tract was removed from three fish and intestinal content squeezed out into individual tubes and placed on dry ice before being stored at − 80 °C for subsequent gut microbiota analysis. The digestive tract was also removed from a further 11 fish per tank and divided into three sections: pyloric caeca (PC), proximal intestine (PI), and distal intestine (DI). Tissue samples for brush border membrane (BBM) enzyme activity and gene expression analysis were collected form each section of five fish per tank and stored individually at − 20 °C or − 80 °C, respectively. Samples from three fish per tank were preserved in a 4% phosphate-formaldehyde buffer (pH = 7.2) for histological purposes. Finally, three fish carcasses were stored at − 20 °C for chemical analysis.

### Analytical methods

The test diets and fillet muscle tissue were analysed for dry matter, crude protein and ash contents according to Hungerford [42]. Total lipid content was measured according to Hungerford [42] modified by Rasmussen and Morrissey [43]. Gross energy content of test diets was measured with an adiabatic bomb calorimeter (IKA C7000, Werke GmbH and Co., Staufen, Germany). The amino acid analyses of the test diets were performed according to Tibaldi et al. [44]. Acid hydrolysis with HCl 6 mol/L at 115–120 °C for 22–24 h was used for all amino acids, except cysteine and methionine, for which performic acid oxidation preceded acid hydrolysis, and tryptophan that was determined after lithium hydroxide (4 mol/L) hydrolysis. The fatty acid methyl esters (FAMEs) of muscle tissue were determined by Varian gas chromatograph CP-3800 GC (ISO 12966-4:2015). FAMEs were identified by comparing the retention time of each FAME component to the standard (Restek Food Industry FAME Mix 35,077). All tests were performed in duplicate.

### Fish growth indices and skin colour analysis

For each dietary treatment, the indicators of growth and body performance were calculated as follows: (1) weight gain (WG) = ((final weight – initial weight) × 1000)/(initial + final weight)/days); (2) specific growth rate (SGR) = ((ln [final weight] – ln [initial weight]) × 100/number of feeding days); (3) condition factor (K) = ([fish weight × 100]/[fish total length^3]); (4) feed intake FI = ((1000 total ingestion)/((final + initial weight)/2))/days)); (5) feed conversion ratio (FCR) = (WG/dry feed intake); (6) protein efficiency ratio (PER) = (weight gain/dry protein intake).

Body colouration patterns were obtained from digital images of 20 fish per treatment using a high-resolution camera (18 real MP) mounted on a support with a lamp (illuminance 4000 K), with fish positioned laterally on the left size. With a focal length of 70 cm between the camera and the fish, colour calibration was performed with ColorChecker (Macbeth ColorChecker) by applying the colorChecker function of the patternize R package [45]. Comparison of colours and their patterns was performed using the patternize [46] and colordistance [47] R packages and by applying the K-means clustering method to extract the dominant colour palette along with its spatial distribution. For more details, see the Additional file 1: Supplementary methods.

### Intestinal histomorphology

After excision of the pyloric ceca (PC), proximal intestine (PI), and distal intestine (DI) parts, sectional samples from six fish per treatment were carefully rinsed with autoclaved seawater to remove residual faeces and immediately fixed in 10% neutral buffered formalin. Samples were routinely processed for histology, sectioned at 3–5 μm thickness. A total of two slides were made from each block. One slide of each treatment was stained with Mayer’s haematoxylin and eosin (HE) or alcian blue/periodic acid-Schiff stain (AB/PAS, pH = 2.5), respectively. After dehydration, slides were cleared in xylene and cover slipped with NeoMount. HE stained slides were used to assess possible degenerative changes and/or inflammation. Slides were examined under the Olympus BX51 light microscope coupled with Olympus DP25 camera. For more details, see the Additional file 1: Supplementary methods.

### Expression analysis of BBM enzyme genes

Total RNA was extracted with TriReagent (Ambion, Austin, Texas, United States) according to the manufacturer’s instructions, dissolved in RNase/DNase-free water (Sigma Aldrich, St. Louis, Missouri, United States) and stored at − 80 °C. Prior to cDNA synthesis, total RNA was treated with 1 unit/μL RNase free DNase I (ThermoFisher Scientific, Waltham, Massachusetts, USA) following the manufacturer’s instructions. cDNA was synthesised from 1 μg total RNA using the High Capacity cDNA Reverse Transcription kit (Life Technologies, Carlsbad, California, United States) according to the manufacturer’s instructions. Real-time PCR was used to analyse the mRNA expression of four BBM: sucrase-isomaltase (SI), peptide transporter 1 (PepT-1), sodium/potassium-transporting ATPase (Na^+^/K^+^ATPase) and aminopeptidase N (APN). The cDNA template was diluted 1:10 in RNase-DNase-free water and each sample was run in duplicate. Real-time PCR was performed using the SYBR™ Green PCR Master Mix (ThermoFisher Scientific) on the Applied Biosystems™ 7500 Real-Time PCR System using previously designed primers [48]. Cycling conditions were as follows: initial denaturation at 95 °C for 5 min, followed by 40 cycles of denaturation at 95 °C for 10 s, annealing and synthesis at 60 °C for 10 s, and final extension at 72 °C for 10 s. β-actin was used as the reference gene for normalisation since its expression level was independent of dietary treatment. Relative expression (represented as arbitrary units) was calculated as the expression of the target gene divided by that of β-actin times 100.

### Brush border membrane (BBM) enzyme activities

The stored gut section of each fish was thawed and diluted 1:10 with iced saline buffer. Each sample was minced in a tissue disruption system (Tissue Lyser II, Qiagen, Hilden, Germany) at 30 Hz for 1 min, centrifuged at 13,500 × *g* for 10 min at 4 °C, and the supernatant stored at − 20 °C until analysis of the BBM enzymes maltase (Malt), sucrase-isomaltase (SI), intestinal alkaline phosphatase (IAP) and leucine aminopeptidase (L-ANP). The activity of Malt and SI was determined according to [49] with slight modifications. Briefly, 20 μL supernatant was added to 20 μL substrate (maltose or sucrose, 0.056 mol/L). After 60 min incubation at 30 °C, the reaction was stopped by placing samples on ice. The analysis was performed in duplicate in a 96-well microplate. Glucose reagent (Sigma-Aldrich, Milan, Italy) was added to the reaction mixture (1:100), and absorbance was determined at 340 nm in a microplate reader (Tecan Sunrise, Mannedorf/Zurich, Switzerland). IAP activity was measured in triplicate using a commercial kit (Paramedical, Pontecagnano Faiano, Sa, Italy) according to the manufacturer’s instructions. The reaction was monitored every 60 s at 25 °C for 3 min, with absorbance measured at 405 nm in a microplate reader (Tecan Sunrise). In order to determine the activity of L-ANP, a solution of 2 mmol/L L-Leucine-p-nitroanilide in DMSO was diluted 1:100 in Tris-HCl 100 mmol/L at pH 8.8. The sample was then added to the solution containing L-Leucine-p-nitroanilide (1:20), and absorbance was monitored every 60 s at 25 °C for 3 min, with absorbance measured at 405 nm in a microplate reader (Tecan Sunrise). The total protein in the supernatant was determined using Bradford reagent and bovine serum albumin (Sigma-Aldrich) as a standard in agreement with the manufacturer. Specific activities of BBM enzymes were expressed as U = μmol/min/mg protein for Malt and SI and mU for L-ANP and IAP.

### Intestinal microbiota 16S rRNA sequencing

Bacterial DNA was extracted from the whole intestine content of 20 experimentally fed European seabass (2 fish per tank, i.e., 4 replicates per treatment) using the Invitrogen PureLink Microbiome DNA Purification kit (Carlsbad, CA, USA) following the manufacturer’s protocol. The commercial services of Microsynth AG (Balgach, Switzerland) were used for library preparation based on Nextera two-step PCR, including purification and pooling. Variable region V4 of the 16S rRNA gene was successfully amplified from 18 DNA extracts using the MiSeq2000 Next Generation system (Illumina, San Diego, CA, USA).

#### Bioinformatics analysis

Microsynth AG (Balgach, Switzerland) performed demultiplexing, removal of adaptors and primers, merging and filtering of trimmed reads, Operational Taxonomic Units (OTUs) building and chimera removal (clustering with 97% identity), and taxonomy assignment (> 60% confidence) based on the Ribosomal Database Project (RDP) [50] using USEARCH [51]. The BIOM (Biological Observation Matrix) file was imported into R (version 4.0.3) using the Phyloseq package [52] and due to variation in sequence depths among samples, rarefaction was performed using the vegan package [53]. Alpha diversity was calculated using richness (number of species/OTUs observed) and the Shannon index. The principal coordinate analysis (PCoA) plot was visualised using Bray-Curtis calculated distance on log-transformed data. OTU circle and Venn diagram were created using MiscMetabar: Miscellaneous functions for metabarcoding analysis [54] and ggplot2 package [55]. The 16S rRNA gene sequences were deposited in the NCBI Sequence Read Archive (SRA) as BioProject PRJNA725685.

### Calculations and statistics

The effects of dietary treatment on growth and body performance indicators, intestinal morphology measured parameters, differences in expression of target BBM enzyme genes, and alpha and beta diversity of the intestinal microbiota were tested by one-way univariate permutational analysis of variance (PERMANOVA) based on similarity matrices calculated using Euclidean distances, and in the case of beta diversity indices, using Bray-Curtis distances. Significant interactions were considered at a *P* level of 0.05. The *P* values were obtained using 999 unrestricted permutations on square-root transformed data, and Monte-Carlo simulation was considered in the case of a low permutation number. A significant PERMANOVA was followed by an appropriate pairwise comparison test to detect differences among treatments. All statistical analyses were performed using the PRIMER 6+ PERMANOVA software package from the Plymouth Marine Laboratory, UK.

BBM enzyme activity data were analysed for normal distribution and homogeneity of variances using the Kolmogorov-Smirnov and Levene’s tests, respectively, and transformed where necessary. To compare means, one-way ANOVA was applied using the IBM-SPSS statistical package (version 24.0.0.1). If appropriate, Duncan’s posthoc test was performed at a 95% significance level. Statistical differences in body coloration areas among treatments were tested by one-way ANOVA and Tukey’s post hoc test. All data were expressed as mean ± SEM.

## Results

### Growth performance, chemical composition, and fatty acid profile of fillet muscle

Fish initially accepted the test diets, and no mortalities were recorded during the trial. At the end of the 147-d experiment, the final body weight of fish was nearly double the initial weight (Table 2, Additional file 1: Fig. S1b). All growth parameters were significantly affected by the dietary treatments. A significant increase in final body weight, daily gain, and SGR was observed in fish receiving the diets VH10 and VH10P30 compared to the other groups. This occurred despite reduced feed intake relative to fish receiving the fish-based diets FH10 and CF (Table 2). The plant-based diet group CV showed the lowest growth values, with no significant difference in feed intake between this group and the groups VH10 and VH10P30. The FCR and PER values with the different feed treatments followed the pattern observed for growth parameters. Similarly, the lowest ratios of FIFO and relative ECR indices were calculated for the VH10 and VH10P30 groups (Additional file 1: Table S1). The effects of dietary treatments on the chemical composition and fatty acid profile of seabass fillet muscle are shown in Table 2. Only crude protein content was significantly affected by the dietary treatment. Fish from the CV treatment had the lowest protein content (%wet weight), though this differed significantly only from the VH10 group (*P* < 0.05). Total lipid, water, and ash content were similar across dietary treatments.

**Table 2:** Growth performance, feed conversion ratio, fillet muscle chemical composition (% of wet weight) and fatty acid profile (% total fatty acids) of European seabass fed test diets over 147 d

Parameter	CV	VH10	VH10P30	FH10	CF	SEM
Body and growth indices						
FBW, g	266.6^a^	312.2^b^	301.3^b^	284.2^c^	285.4^c^	5.041
K	1.17^a^	1.30^b^	1.25^c^	1.24^c^	1.25^c^	0.012
WG, g/kg/d	2.99^a^	4.31^b^	3.95^b^	3.69^ab^	3.73^b^	0.163
SGR	0.38^a^	0.50^b^	0.47^b^	0.44^a^	0.45^a^	0.013
FI, g/kg/d	6.60^a^	6.45^a^	6.59^a^	7.60^b^	7.23^ab^	0.182
FCR	1.87^a^	1.32^b^	1.40^b^	1.72^ac^	1.63^c^	0.069
PER	1.19^a^	1.67^b^	1.57^b^	1.27^ac^	1.35^c^	0.623
Fillet muscle chemical composition, %						
Water	70.7	69.3	70.0	70.4	70.3	0.23
Lipids	6.9	7.4	6.8	7.0	7.2	0.20
Crude protein	20.5^a^	22.0^b^	21.5^ab^	21.1^ab^	20.7^ab^	0.14
Ash	1.3	1.2	1.3	1.2	1.3	0.01
Fatty acid profile, % of total FA						
16:0	17.3^ab^	16.4^a^	18.3^ab^	19.8^c^	19.7^c^	0.48
18:0	3.7^a^	3.9^ab^	4.0^b^	4.1^b^	4.0^b^	0.05
SFA	26.0^a^	24.7^ab^	27.6^a^	30.0^c^	29.2^c^	0.72
16:1n-7cis	4.9^a^	4.2^a^	5.1^ab^	5.9^b^	5.8^b^	0.23
18:1n-9 cis	29.8^a^	35.3^b^	30.0^a^	27.3^c^	27.0^c^	0.96
20:1n-9	1.9^a^	2.0^a^	2.1^a^	2.8^b^	2.7^b^	0.13
MUFA	37.5	41.2	38.0	37.6	36.7	0.65
18:2n-6 cis	16.4^a^	15.2^b^	15.2^b^	11.8^c^	12.2^c^	0.62
20:2n-6	1.1	1.2	1.2	1.3	1.2	0.02
n-6PUFA	17.5^a^	16.7^ab^	16.5^b^	13.3^c^	13.6^c^	0.61
18:3n-3	6.5^a^	6.6^a^	5.8^b^	4.3^c^	4.8^c^	0.32
20:5n-3	6.1^a^	5.3^b^	6.3^a^	7.5^c^	7.4^c^	0.30
22:6n-3	6.0^a^	4.9^a^	6.0^a^	7.6^b^	7.2^b^	0.36
n-3PUFA	18.0^a^	17.0^b^	18.1^a^	19.4^a^	19.3^a^	0.33
n-3/n-6	1.04^a^	1.02^a^	1.10^a^	1.46^b^	1.42^b^	0.07

Initial mean body weight (± SEM) of 149 ± 1.04 g was not significantly different among treatment groups (*P* > 0.05). *FBW* final body weight, *K* condition factor, *WG* weight gain, *SGR* specific growth weight, *FI* feed intake, *FCR* feed conversion rate, *PER* protein efficiency ratio. Proximate composition and fatty acid profile values are presented as mean (*n* = 3) and pooled standard error of the mean (± SEM). Row means indicated with different superscript letters are significantly different (*P* < 0.05)

The fatty acid profile of fillet muscle varied significantly among dietary treatments (Table 2). Fish receiving fish-based diets (FH10 and CF) had a significantly higher percentage of SFA than fish fed diets with a higher plant component (CV, VH10, VH10P30; Table 2), mainly due to the increased proportion of palmitic acid. Regarding MUFA, oleic acid (18:1n9) was the predominant fatty acid in fish groups and was significantly (*P* < 0.05) higher in all groups fed CV-based diets than in those receiving fish-based diets. The highest proportion of oleic acid was recorded in the VH10 group, which received 10% crude protein from *H. illucens* pupae meal. However, the contribution of total MUFA did not vary significantly among groups. Finally, the contribution of dietary plant lipid sources resulted in a significant increase in n-6 PUFA in muscle fillet and, at the same time, in a slight but non-significant decrease in n-3 PUFA in fish receiving the diets CV, VH10 and VH10P30 compared to those given diets FH10 and CF. Accordingly, the n-3/n-6 ratio was significantly (*P* < 0.05) increased in fish in the FH10 and CF groups compared to the other dietary groups. Linoleic acid (18:2n6) was the most abundant PUFA in muscle lipids, regardless of dietary treatment. It showed increased levels of up to 30% in fish receiving plant-based dietary treatments, which significantly affected the overall n-6 PUFA status among the treatments. The opposite situation was found for eicosapentaenoic (20:5n3, EPA) and docosahexaenoic (22:6n3, DHA) acids, whose proportions were significantly higher in the muscle of fish fed fish-based diets, although changes in total n-3 PUFA among dietary groups was less pronounced than for total n-6 PUFAs.

### Skin colouration

**Fig. 1:**
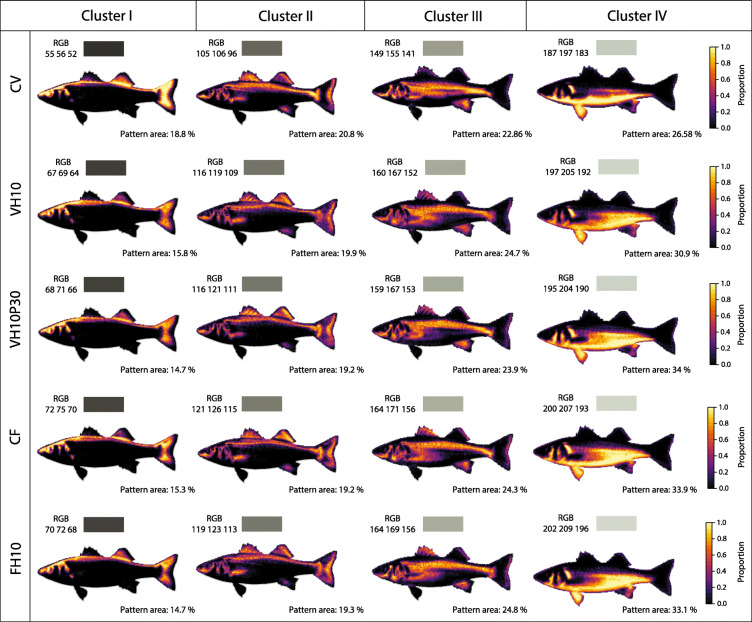
Image registration and k-means clustering of the colour patterns of European seabass. For treatment groups (rows), colours were k-means clustered into four groups (columns), ranging from dark to light grey colour hues with displayed average RGB values and coverage (%) of extracted colour pattern area on the body surface

Skin colour expression varied significantly among the five groups of fish in response to different dietary treatments, extracting four colour clusters ranging from dark to light grey hues. The histogram of pairwise colour distance scores (CDS) showed that the CV group had the most uniform colour distribution among individuals compared to the other groups (Additional file 1: Fig. S2). Overall, CDS varied significantly among treatments (F = 71. 81, *P* < 0.01), with only the FH10 vs. VH10 comparison showing no significant differentiation (*P* = 0.425). Heatmap visualisation of colour patterns showed that the CV group had the dullest coloration, with darker shades of grey (lowest RGB values) compared to the lighter silver-grey body coloration of the other treatments (Fig. 1). The first and fourth clusters of CV had the highest proportions, with conspicuous pattern distributions on the posterior area of the operculum, head profile, dorsal-ventral body region, and pectoral and caudal fin. Although the other treatment groups had similar RGB values and colour pattern coverages (%), slight discrepancies among them resulted in a partitioning of grey coloration by each colour cluster, as shown in the RDA plot (Additional file 1: Fig. S3). A significant difference in the pattern areas of the clusters was only found when comparing CV with other treatments.

### Intestinal morphology

**Fig. 2:**
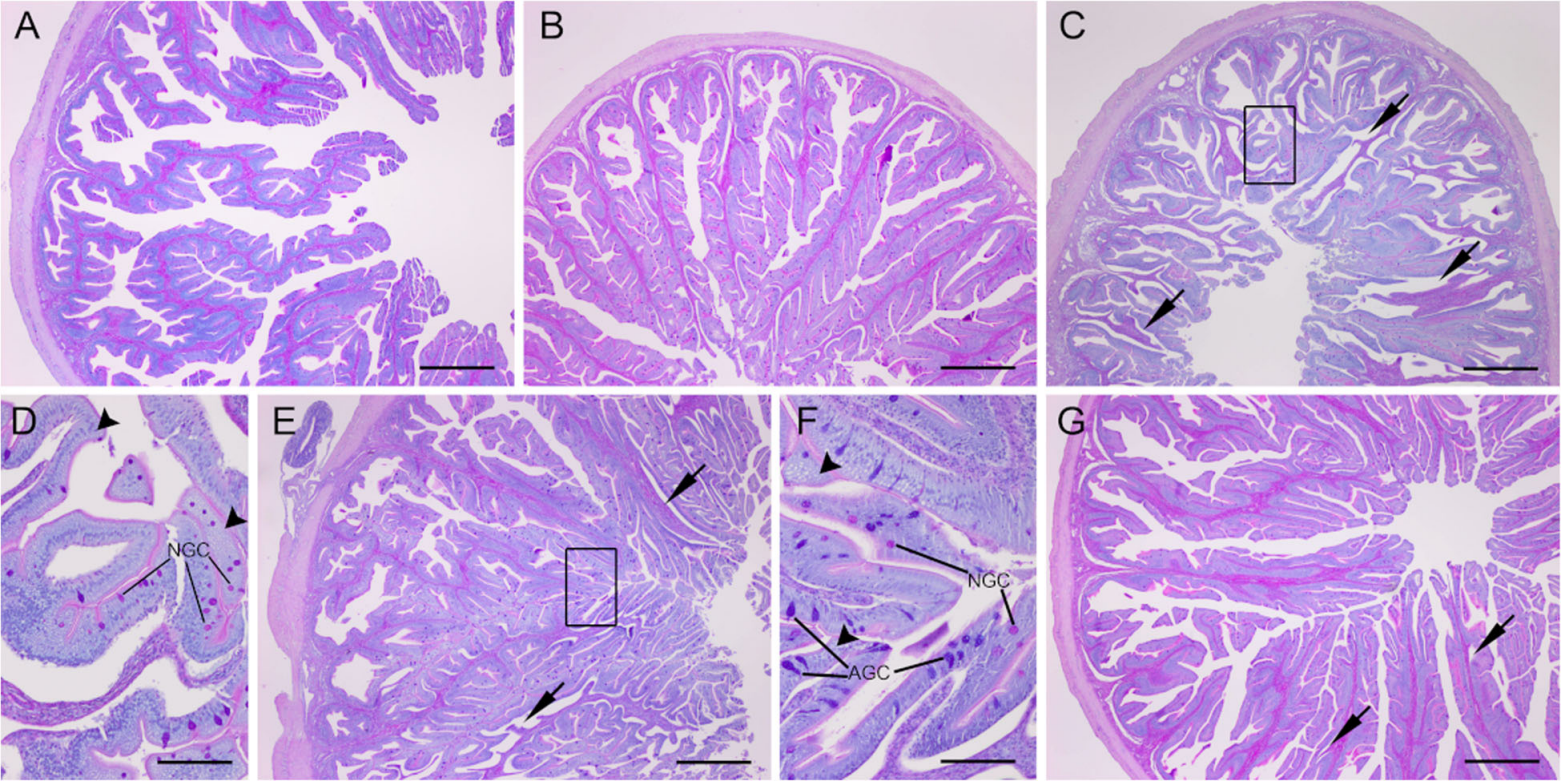
Proximal intestine histological sections of fish fed different test diets. **A** CF diet, well preserved intestinal morphology with elongated and regularly branched complex villi. **B** FH10 diet, similar to the CF diet, intestinal morphology was well preserved with elongated and regularly branched complex villi. **C** CV diet, elongated, irregularly branched complex villi with extensive multifocal detachment of lamina epithelialis from the lamina propria (arrows). **D** Detail of area indicated by the rectangle in panel C; note the pronounced vacuolisation of enterocytes (arrow heads) and notable number of neutral goblet cells (NGC). **E** VH10 diet, elongated, irregularly and highly branched complex villi with multifocal moderate to extensive detachment of lamina epithelialis from the lamina propria (arrows). **F** Detail of area indicated with by the rectangle in panel E; note the moderate vacuolisation of enterocytes (arrow heads) with the predominance of acid goblet cells (AGC) over neutral goblet cells (NGC). **G** VH10P30 diet, elongated and irregularly branched complex villi with multifocal moderate to extensive detachment of lamina epithelialis from the lamina propria (arrows). Alcian blue/PAS staining, pH = 2.5. Scale bar: A, B, C, E, G = 500 μm, D, F = 100 μm

Due to poor embedding of many samples, the pyloric caeca were excluded from the morphometric analysis. All other intestinal sections from all sampled fish were included in the analysis, with no samples discarded. In the proximal and distal intestines, all morphometric parameters varied significantly among dietary groups (Additional file 1: Table S2). However, the pairwise comparison showed a different significance level for each parameter between pairs of experimental groups. In fish fed diets high in fish-derived protein (CF and FH10), the proximal intestine showed a well-preserved morphology with elongated, regularly branched complex villi, no signs of enterocyte vacuolisation and only focal fusion of the simple villi (Fig. 2a-b). In both these experimental groups, the lamina propria was populated with eosinophil granule cells (EGCs), and few lymphocytes were detected infiltrating the lamina propria or lamina epithelialis. In fish fed diets high in plant-derived protein content (CV and VH10), the complex villi were elongated, irregularly branched with extensive enterocyte vacuolisation, and were more pronounced in fish fed the diet without the addition of insect crude protein (Fig. 2c-d). Both of these experimental groups exhibited focal detachment of the lamina epithelialis from the lamina propria, extending over a considerable length of the villi. More pronounced infiltration of EGCs in the lamina propria and submucosa was seen, as well as moderate to abundant lymphocyte infiltration in the lamina propria and lamina epithelialis. In fish fed a diet with the addition of PBM, the villi were elongated, irregularly branched with minor focal enterocyte vacuolisation and fusion of lateral branches (Fig. 2e). Focally extensive detachment of the lamina epithelialis from the lamina propria was noted, with mild to moderate lymphocyte infiltration in both the lamina propria and lamina epithelialis. Less pronounced degenerative changes were observed in the distal intestine, mostly limited to the overall appearance of the villi. Villi fusion was observed in all experimental groups, but was more pronounced in the groups fed a plant-based diet, likely due to the fusion of more than two adjacent villi (Additional file 1: Fig. S4). Additionally, moderate detachment of the lamina propria from the lamina epithelialis was noted in groups fed a plant-based diet. Mild lymphocyte infiltration in the lamina propria and lamina epithelialis was present in these experimental groups, whereas the other experimental groups showed no evidence of inflammatory changes.

### Expression of BBM enzyme genes

**Fig. 3:**
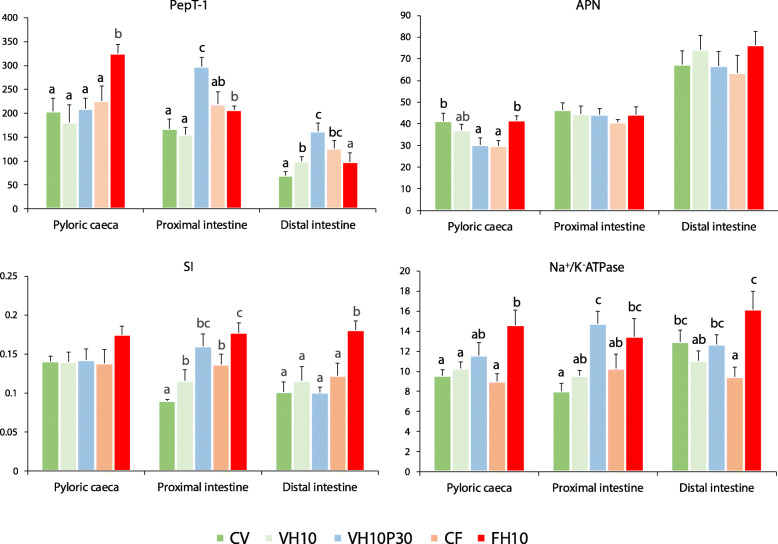
Relative expression of genes encoding BBM enzymes in European seabass intestine. Relative expression of genes encoding BBM enzymes peptide transporter 1 (PepT-1), aminopeptidase N (APN), sucrose-isomaltase (SI) and sodium/potassium-transporting ATPase (Na^+^/K^+^ATPase) in European seabass pyloric caeca, proximal and distal intestine at the end of the 21-week feeding period. The relative expression (arbitrary units) was calculated as the expression of the target gene divided by that of β-actin multiplied by 100. Data are presented as the average + SEM of eight fish per group. Different letters indicate significant differences among dietary treatments (*P* < 0.05)

Analysis of BBM enzyme gene expression (Fig. 3) revealed that each dietary treatment differentially affected BBM enzyme gene transcription in different parts of the intestine, with the diets FH10 and VH10P30 causing the highest induction of most BBM genes. PepT-1 was significantly upregulated in the FH10 group compared to other treatments, but only in PC. In PI and DI, PepT-1 showed the highest induction in the H10P30 group. Furthermore, an overall decrease in PepT-1 expression towards the distal part of the intestine was noticed in all fish groups (Fig. 3, PepT-1). APN was significantly upregulated in the VH10P30 and FH10 groups compared to other groups, but only in PC. APN transcription was not affected by dietary treatment changes in PI and DI in any group. However, an increase in APN expression level towards the distal part of the intestine was observed in all groups (Fig. 3, APN). Changes in diet formulation resulted in no differences in SI expression between groups, only in PC. In PI, SI was significantly upregulated in the FH10 group compared to the other groups, except for VH10P30. Similarly, in DI, there was a statistically significant difference in the expression of SI between the FH10 group and all other groups (Fig. 3, SI). Na^+/^K^+^ATPase was significantly upregulated in PC of the FH10 group compared to other groups, except VH10P30. In PI, the Na^+^/K^+^ATPase gene was significantly upregulated in the VH10P30 group compared to all groups except FH10. In DI, statistically significant up-regulation was found in the FH10 group, but only in regard to the group fed with control diet CF (Fig. 3, Na^+^/K^+^ATPase).

### BBM enzyme activities

**Fig. 4:**
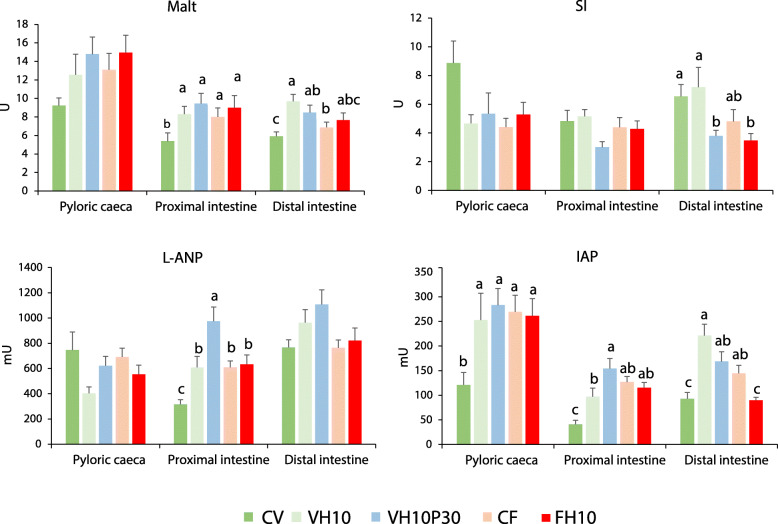
Activity of the BBM enzymes in the European seabass intestine. Activity of the BBM enzymes maltase (Malt), sucrase (SI), leucine-aminopeptidase (L-ANP) and intestinal alkaline phosphatase (IAP) in European seabass fed the experimental diets over 21 weeks. Different letters indicate significant differences among dietary treatments (*P* < 0.05)

Malt specific activity showed a similar pattern in the three intestinal sections, although in PC the differences among treatments were not statistically significant, likely due to the high variability of samples (Fig. 4, Malt). In the PI of fish fed a diet with higher plant content (CV), Malt activity decreased significantly compared to the other groups. In the distal part of the intestine, fish fed with CV diet showed the lowest levels of Malt, while the VH10 and VH10P30 treatments increased the Malt activity. The specific activity of SI was affected by treatment only in DI, where the CF, CV, and VH10 groups showed the highest SI activity (Fig. 4, SI). The activity of the protease L-ANP was affected by the treatments only in the PI (Fig. 4, L-ANP), where it was the highest in the VH10P30 group and significantly different from all the other treatments. Fish in the CV group showed significantly lowest L-ANP activity, while those in the CF, FH10, and VH10 groups showed a significant increase. The IAP was affected by dietary treatments in each intestinal tract (Fig. 4, IAP). The activity was statistically the lowest in PC and PI in the CV group. While there was no statistical difference in IAP activity between the other groups in the first part, activity in the proximal part was statistically the highest in the VH10P30 group. Finally, the plant-based diet containing insect meal alone (VH10) or with PBM (VH10P30) triggered the highest activity in the DI of the tested fish. In general, the addition of insect meal to fish meal (FH10) did not affect the activity of BBM enzymes, while the substitution of the plant mixture with insect meal alone (VH10) or with poultry by-products (VH10P30) differently modified their activities.

### Intestinal microbiota composition

**Fig. 5:**
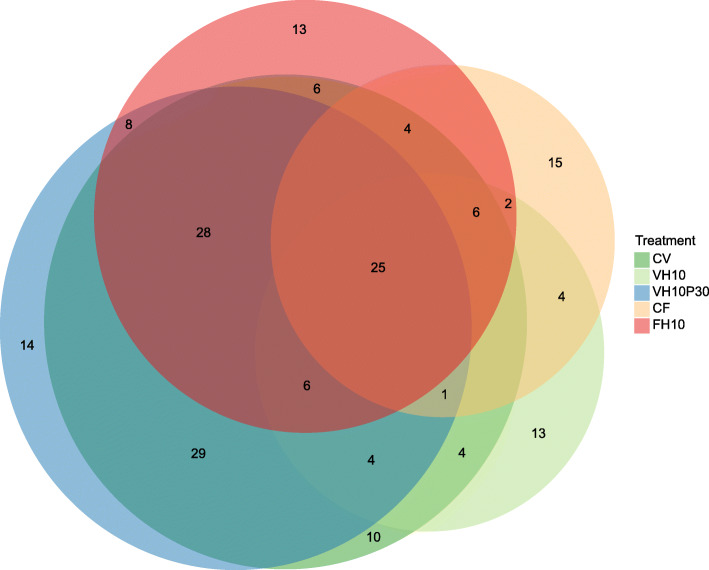
Venn diagram. Venn diagram depicting unique and shared OTUs between treatments, after restricting the minimal number of sequences to 5, using MiscMetabar: Miscellaneous functions for metabarcoding analysis (https://github.com/adrientaudiere/MiscMetabar)

**Fig. 6:**
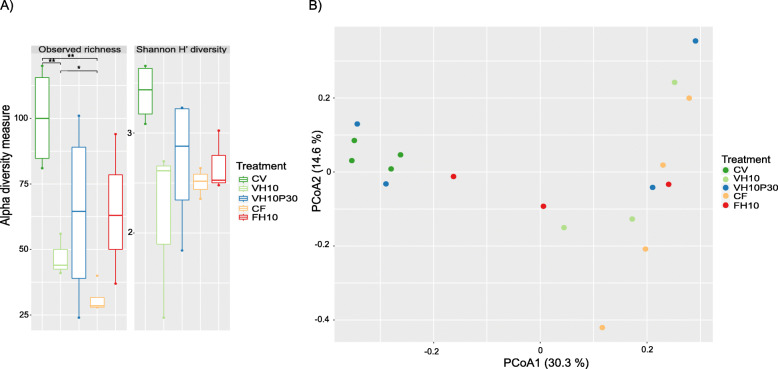
Alpha and Beta diversity. **A** Alpha diversity was assessed using observed richness and the Shannon H′ diversity index. Data are shown as boxplots (4 or 3 replicates per treatment). *P*-values indicate significant difference between treatments, * *P* < 0.05, ** *P* < 0.01. **B** Beta diversity was visualised using Principal Coordinates Analysis (PCoA), based on Bray Curtis distance and log-transformed data. Significant differences were detected between the CV and VH10 treatment, and CV and CF treatment, respectively (*P* < 0.05) (data available in Additional file 1: Fig. S4)

A total of 769,200 quality-filtered reads corresponded to an average of 42,732 reads per sample (SD ± 18,111 reads). Overall, in 18 sequenced samples of the whole microbial community in the intestine of experimentally fed subadult European seabass, 205 OTUs were observed. The Venn diagram depicting unique and shared OTUs between treatments demonstrated the greatest overlap between the groups CV and VH10P30; and CV, VH10P30, and FH10, respectively. The CF group had the highest number of unique OTUs (Fig. 5). The most pronounced differences in observed richness were found between the groups CV and VH10 (*P* < 0.01), CV and CF (*P* < 0.01), and VH10 and CF (*P* < 0.05), respectively (Fig. 6A, Additional file 1: Table S3). However, no differences were detected between groups according to the Shannon H′ index of diversity (Fig. 6A). Principal Coordinates Analysis (PCoA) of β-diversity revealed a consistent pattern of interactions as found in the observed richness (Fig. 6B). A significant difference was observed between the groups CV and VH10 (*P* < 0.05), and CV and CF (*P* < 0.05) (Additional file 1: Table S4).

**Fig. 7:**
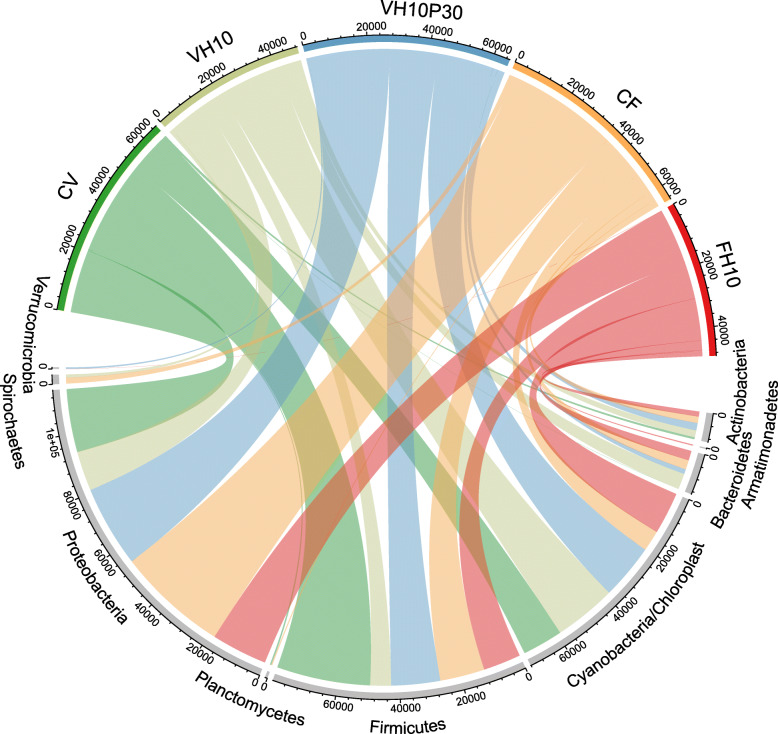
Bacterial community in subadult European seabass intestine. OTU circle (produced using MiscMetabar: Miscellaneous functions for metabarcoding analysis, https://github.com/adrientaudiere/MiscMetabar), connecting each feeding treatment with the phylum taxonomic level (nine most represented) of the bacterial community determined in the intestinal content of subadult European seabass

**Fig. 8:**
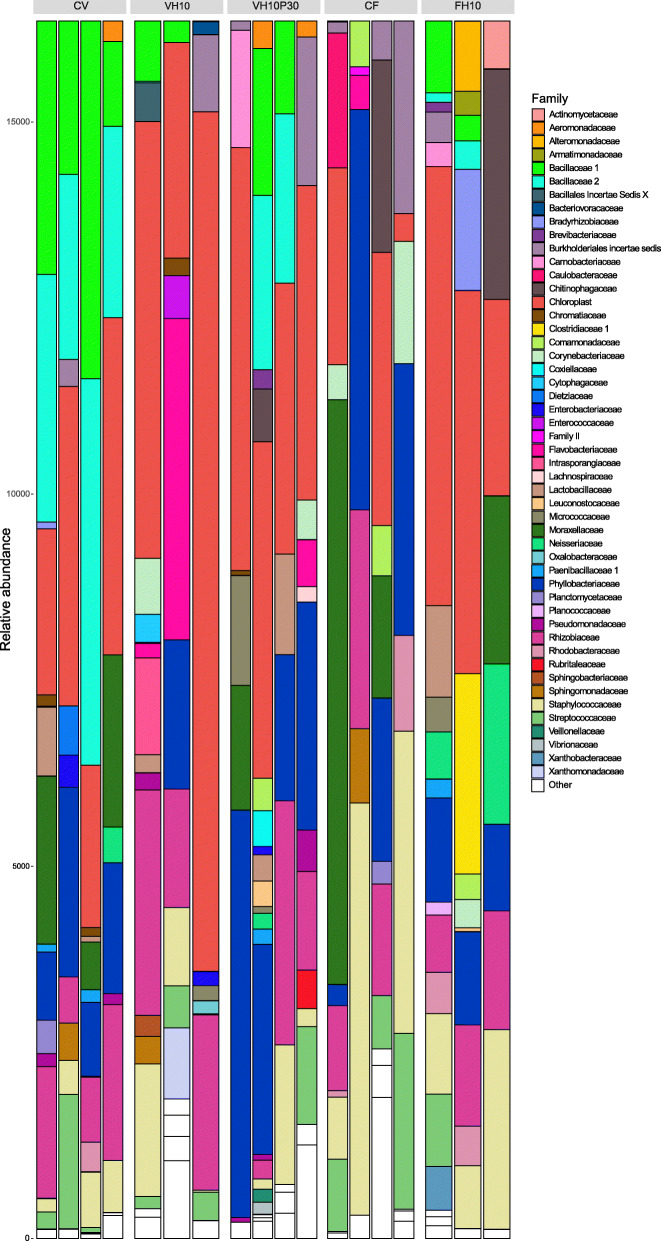
Relative abundance of European seabass microbiome. Subadult European seabass microbiome relative abundance barplot at the family taxonomic level, according to feeding treatment. Color-coded family legend is given next to the barplot

The total microbial community of the 18 intestinal samples was mainly composed of nine phyla, with the majority of the constituents belonging to the phyla Firmicutes, Proteobacteria, Actinobacteria, Bacteroidetes, and Cyanobacteria/Chloroplast (Fig. 7). It is important to note that phylum Bacteroidetes was not detected in the CV group, while it was present in at least one biological replicate of all other treatments, with a more pronounced difference compared to the groups VH10 and CF. Furthermore, the phylum Spirochaetes was present in the groups VH10 and CF, but not in CV. Based on the intestinal microbial community profiles for each dietary treatment and biological replicates at the taxonomic family level (Fig. 8), it can be observed that the family Bacillaceae dominated in the CV group and was frequent in VH10P30, while it appeared less frequently in the other groups.

## Discussion

In the attempt to develop alternative fish feeds for more sustainable aquaculture, five experimental diets were formulated to meet the nutritional needs of subadult European seabass. To our knowledge, this is the first study focused on both plant-based and FM-based diets for subadult seabass, where the plant-based ingredients are partially replaced by insect meal (i.e., *H. illucens* pupae meal), alone or in combination with PAPs (i.e., poultry by-product meal). Although there are several studies [26, 28, 56], the inclusion of *H. illucens* meal or PBM as a substitution for FM in diets for seabass, and the combination of the two as a supplement to a plant-based diet, has not yet been investigated. Finally, this is the first study to comprehensively evaluate the effects of an alternative dietary treatment for seabass.

### Effect of experimental diets on seabass growth performance, chemical composition, and fatty acid profile

The results of the present study demonstrate that a plant-based feed enriched with 10% crude protein from commercially defatted *H. illucens* pupae meal alone (VH10) or in combination with 30% PBM (VH10P30) significantly increased the final body weight and SGR of subadult seabass compared to the CV diet and, interestingly, even to the FM-based diets CF and FH10. Our results are consistent with previous studies evaluating PBM as a protein source for partial substitution of FM, yielding a similar growth performance to that obtained with FM for seabass [56] and other fish species [57–62]. Furthermore, the beneficial effect of *H. illucens* on the growth performance of seabass, as shown in the present study, confirm previous findings on the successful partial substitution of FM with *H. illucens* and other insect meals [63–65]. A comprehensive meta-analysis on animal protein sources in aquaculture also pointed out the great potential of poultry by-products and insect meal as efficient substitutes for FM in the diets of various fish species and size groups, with positive effects on growth rate and FCR [4]. Interestingly, although a significant increase in voluntary feed intake was attributed to the fish-based diets (FH10 and CF groups), this was not reflected in a better growth rate. The higher feed intake in the FH10 and CF groups can be explained by high sensitivity to tastants, and consequently by the choice of raw ingredients and the attractiveness of test diets with respect to their chemical cues [66, 67], as this became more pronounced with a decrease in temperature. Although seabass has shown to have relatively good tolerance to diets containing high levels of soybean meal [4, 10], a possible negative effect of antinutrients derived from different plant origin in the CV diet on growth performance cannot be completely ruled out. Both the VH10 and VH10P30 diets had a sustained fish-in fish-out ratio less than 1.0 (0.67–0.75) and this was supported by the increase in feed conversion efficiency. Similarly, these diets were economically competitive and the most cost-effective compared to diets based on FM. In the present study, proximate muscle composition of the European seabass was not strongly affected by dietary treatments, but the opposite was found for fatty acid composition, reflecting the different profiles of the dietary oils used. Using plant oil as an alternative to fish oil affects the fatty acid composition of fish [68–71], especially in marine fish species, because of their limited ability to convert 18C fatty acids to 20–22C fatty acids in comparison with freshwater fish [72]. All fish groups receiving about 66% plant oils (CV, VH10 and VH10P30) had significantly higher levels of n-9 and n-6 fatty acids, and slightly lower levels of n-3 fatty acids compared to the fish groups receiving FO (FH10, CF), which was expected since most plant oils are rich in n-6/n-9 fatty acids and poor in n-3 fatty acids compared to marine oils [73]. Partial substitution of up to 60% dietary FO with various plant oils without negative effects on fish growth performance has already been achieved [74–76], but with side effects that generally affect the deposition of linoleic acid (18:2n-6) and levels of n-3 polyunsaturated fatty acids such as EPA and DHA in the fillet. In the present study, muscle concentrations of EPA and DPA were reduced by only 15–19% in the plant oil-fed treatment groups compared with the FO-fed groups, and although the n-3/n-6 ratio was significantly lower in the plant lipid groups, it was still within the WHO/FAO [77] recommendations. Siddik et al. [78] and Fabrikov et al. [79] recently demonstrated that n-3 fatty acids in fish fillets are significantly reduced by the inclusion of poultry by-products and insect meal in FM-based diets for various fish species. However, it must be emphasised that the amounts of added poultry by-products and insect meal were higher than in the present study, ranging from 75% to 100% and 15–30%, respectively, indicating the importance of achieving an optimal ratio of all ingredients included in the diet. As for the PUFA:SFA ratio, the overall values in all treatment groups were higher than the minimum recommended value of 0.45 [80], supporting the positive assessment of the nutritional quality of all fish groups.

### Effects of dietary treatment on seabass intestinal histomorphology

Dietary treatment had a significant effect on morphometric parameters and overall intestinal morphology, albeit with different significance levels. In comparison, increased levels of plant meal and oils had no significant impact on the intestinal morphology of juvenile seabass [8], though in the present study, more significant degenerative changes were observed in PI than in DI, with the expectedly better preservation of PI morphology in fish receiving the CF and FH10 diets. Similar findings were also reported by Basto et al. [65] for seabass fed a diet supplemented with defatted *T. molitor* larval protein, where no evidence of villous fusion or enterocyte vacuolisation was found. Conversely, detachment of the lamina epithelialis from the lamina propria and increased supranuclear enterocyte vacuolisation in fish fed diets with increased levels of plant components was recorded here, but with no apparent lesions of the epithelial barrier. The former is probably a processing artefact, i.e., likely caused by the friction during sectioning, yet it indicates impaired integrity of the intestinal wall. Vacuolisation of enterocytes due to ingestion of plant components has been reported in several fish species, affecting different parts of the intestine [81–83]. Similar to the present findings, no studies reported damage to the epithelium associated with increased vacuolisation of enterocytes. The presence of absorptive vacuoles in the epithelial layer occurs frequently, but their excessive number could affect the normal function of enterocytes or even cause damage to cells [84]. Moreover, increased vacuolisation is often accompanied by increased leukocyte infiltration, as reported in several species fed diets high in plant protein or containing grated levels of soybean meal, dietary probiotics or microalgae [83, 85, 86]. Indeed, increased infiltration of eosinophil granule cells (EGCs) and lymphocytes in the lamina propria and lamina epithelialis in fish receiving the plant-based diet was observed here. Combined with increased enterocyte vacuolisation, these inflammatory changes were similar to soybean meal-induced enteritis (SBMIE) reported in Atlantic salmon [87]. However, PBM supplementation appeared to mitigate these effects to some extent. Cellular infiltration was less pronounced, enterocytes exhibited a lower degree of vacuolisation, and even distal intestinal morphometric parameters were comparable to those of the control diet. Nevertheless, in barramundi (*Lates calcalifer*), a complete substitution of FM with PBM negatively impacted intestinal structure at the light microscopy and ultrastructural level [88], suggesting that PBM could replace FM to some extent. Discrepancies with previous studies are probably related to different species and ages, trial duration, feeds used, and processing methods resulting in different nutrient profiles [65], but also reflect the different tolerance of species to antinutrients in plant-based feeds.

### Changes in the expression of BBM genes in response to dietary treatments

The impact of different diet formulations on the expression of BBM enzyme genes has already been confirmed in several teleost fishes [49, 89–93]. However, the mechanisms underlying these changes are still not fully elucidated. In the present study, transcription of BBM enzyme genes varied significantly in response to dietary treatment in different parts of the European seabass intestinal tract. In general, the tri- and di- peptide transporter PepT-1 is primarily expressed in the pyloric caeca and proximal intestine of fish, with a marked decrease in expression towards the distal intestine [94]. This is consistent with the present results where lower levels of PepT-1 transcripts were detected in all fish groups in the DI. In higher vertebrates, PepT-1 shows increased expression in response to a high-level and/or high-quality protein diet [95–97]. On the other hand, studies on the effect of dietary treatment on PepT-1 expression in the fish intestine yielded contradictory results. For example, PepT-1 expression in Asian weatherloach (*Misgurnus anguillicaudatus*) and Atlantic cod (*Gadus morhua*) larvae was not affected by protein content or source in the diet, respectively [98, 99]. In contrast, changing the protein source altered intestinal expression of Pept-1 in alevins of rainbow trout (*Oncorhynchus mykiss*) [92] and Atlantic cod [100]. The present study also showed that the choice of protein source affected PepT-1 expression in all three intestine segments of seabass. This supports the assumption that PepT-1 is highly responsive in fish and its expression varies with multifarious dietary treatments [94]. Changes in protein source did not affect the expression of APN, an enzyme involved in the absorption of dietary proteins [101], in the proximal and distal parts of the seabass intestine, suggesting that APN responds to changes in protein content rather than the source itself, as shown previously in grass carp (*Ctenopharyngodon idella*) [102] and even in some higher vertebrates [103]. APN was most highly expressed in the distal part of the seabass intestine, as opposed to previous findings in grass carp [102] and some higher vertebrates [104], where expression decreased along the intestine. SI is a glucosidase enzyme responsible for the digestion and absorption of dietary carbohydrates such as starch, sucrose and isomaltose [105]. Interestingly, it was upregulated along the entire PI and DI of fish fed the FH10 diet. One would expect the highest induction of this BBM enzyme in fish fed the control CV feed or VH10, due to their high plant-derived content. However, these plant components are mainly protein concentrates and gluten, the latter containing relatively high protein but low starch [106]. Furthermore, the feed formulations of CV and VH10 have a much lower portion of wheat meal and whole peas than FH10. Since sucrose is the main transport sugar available for starch synthesis in pea, accounting for 95% of total pea sugar [107], and starch is the main carbohydrate component in wheat [108], this could explain the highest induction of SI in the fish fed FH10. Na^+^/K^+^ATPase is crucial for maintaining intracellular homeostasis by regulating osmotic balance, cell volume, cytoplasmic pH, Ca^2+^ levels, and Na^+^-coupled transport of nutrients and amino acids into cells [109]. Similar to SI and PepT-1, the highest induction of the Na^+^/K^+^ATPase gene was measured in the FH10 and VH10P30 groups. This suggests that dietary manipulations with different protein sources can affect Na^+^/K^+^ATPase expression in fish and consequently cell homeostasis, as previously reported in fasted/refed seabass [110] and seabass fed a microalgae-enriched diet [48]. Test diets containing *H. illucens* or administered in combination with PBM had beneficial effects on BBM enzyme gene activity and potentially improved digestive/absorption functions compared to fish fed a plant-based diet.

### Effect of dietary treatments on BBM enzyme activity

In the present study, BBM enzyme activity was assessed 24 h after the last meal, giving insight into the long-term adaptability of fish to diets, rather than the response that occurs within hours of the meal [111]. Similar to the expression of the gene SI, the decreased activity of Malt in PI and DI in the CV group appeared to reflect the lower starch content of the CV diet compared to the CF diet. The plant-based diets containing *H. illucens* meal alone or with PBM also contained a higher proportion of wheat meal than the CV diet, which probably stimulated the activity of Malt. Sucrase activity did not appear to be affected by the amount of dietary starch ingested. It is not clear whether and how *H. illucens* meal and PBM may affect sucrase activity in the distal intestine. In addition, a possible positive effect of chitin in insect-containing diets on the activity of disaccharidases could be hypothesised. Krogdahl et al. [112] investigated the effects of different dietary starch contents on the activity of Malt and sucrase in Atlantic salmon (*Salmo salar*) and rainbow trout. Those authors further reported significant differences between species and a positive correlation between starch content and intestinal activity of disaccharidases. On the contrary, Tibaldi et al. [10] found no correlation between dietary starch intake and Malt activity in seabass.

The activity of L-ANP in the brush border membrane provides information on the extent of protein digestion. Substitution of the fish component with the plant protein source in the diet significantly affected L-ANP only in the proximal intestine, where the bulk of protein digestion occurs. Partial replacement of plant-based meal with *H. illucens* meal stimulated the activity of this enzyme and reached levels similar to those of the diet containing fish meal. In a recent work on Atlantic salmon, Belghit et al. [113] reported a negative effect of replacing 85% of protein from FM with *H. illucens* meal in the proximal and midgut due to the chitin content in HM feeds. The presence of a certain amount of chitin could disrupt intestinal homeostasis and cause changes in intestinal turnover or exfoliation. However, in this study, plant-based ingredients were replaced by only 10% *H. illucens*-derived crude protein, suggesting that the low amount of chitin (4.8% in HM) could positively affect protein digestion of seabass. Moreover, the inclusion of PBM in the plant diet greatly increased the activity of L-ANP. Thus, it seems that the proximal intestine is more responsive to the quality of the protein source in the diet than the distal intestine and pyloric caeca, reflecting the final body weight, specific growth rate, and protein efficiency ratio of the fish receiving the plant-based diets (CV, VH10 and VH10P30). This suggests that protein digestion in seabass may impact zootechnical performance.

IAP is a hydrolase enzyme that plays an essential role in maintaining intestinal homeostasis and health. It regulates gut microbiota by promoting bacterial colonisation with commensal organisms, prevents intestinal inflammation by inactivating microbial lipopolysaccharide (LPS), and controls nutrient absorption (e.g., calcium, phosphorus, fatty acids) [114, 119]. In our study, the substitution of fish meal with plant-based ingredients, mainly soy protein concentrate and wheat meal, had a negative effect on IAP activity, as previously reported in Atlantic salmon [115] and European seabass [10]. In contrast, soybean products had no or transient effects on IAP activity in Atlantic cod [116], juvenile drum (*Totoaba macdonaldi*) [117] and gilthead seabream [118]. Since IAP is expressed by enterocytes [15, 119], the low activity of IAP could be due to impaired function of enterocytes associated with their increased vacuolisation, which was observed in the histological analysis of the seabass intestine. Interestingly, adding a small amount of *H. illucens* meal (8%) to both FM- and plant-based diets stimulated a significant increase in IAP activity. This stimulatory effect is maintained when PBM is added, reflecting the results of the histological analysis, which showed a lower degree of enterocyte vacuolisation in this fish group. The present data agree with those of Hekmatpour et al. [120] who reported a stimulatory effect of replacing FM with 35% and 45% PBM in the diet of sobaity seabream (*Sparidentex hasta).* In contrast, different percentages of PBM inclusion did not affect the IAP activity of gilthead seabream [121].

### Effect of experimental diets on seabass intestinal microbiota composition

Recently, research in nutritional manipulation in aquaculture has been focused on modifications in gut microbiota as an indicator of fish health and welfare [122]. The gut microbiome has previously been confirmed to influence fish metabolism, and regulate nutrient uptake [123] and metabolic pathways [124]. Functional flexibility of the microbiome likely plays an important role in the digestive adaptability of the fish [125]. In the current study, 16S rRNA sequencing revealed that European seabass from each treatment group had different gut microbial communities. It is important to note that the groups receiving defatted *H. illucens* pupae meal (VH10, VH10P30, and FH10) had higher bacterial community richness and reduced Proteobacteria compared to the CF treatment. The same pattern was observed in studies on juvenile rainbow trout [126, 127] and pikeperch (*Sander lucioperca*) [128], investigating the effects of insect meal from *H. illucens* larvae on the gut microbiota. The authors concluded that beneficial changes in the composition of the gut microbiota caused by the ingestion of insect meal, such as an increased presence of lactic acid bacteria, could be attributed to the prebiotic properties of fermentable chitin [126, 127]. The inclusion of plant proteins, poultry by-products, and insect meal in this study (CV, VH10P30, and FH10) also increased the abundance of the phylum Firmicutes, which is especially relevant for the Bacillaceae and Lactobacillaceae families that were not detected in the control CF diet (Fig. 8). Beneficial genera, such as *Lactobacillus* and *Bacillus*, were increased in the intestine of seabass in the CV, VH10P30, and FH10 groups (Additional file 1: Fig. S4). These genera were also enriched in the gut microbial communities of subadult rainbow trout fed *H. illucens* insect meal in combination with a low FM base [129]. Another study on zebrafish (*Danio rerio*) experimentally fed with five diets with increasing percentage of *H. illucens* full fat prepupae meal suggested that compensatory enrichment of some taxa might occur in the gut microbiota in order to maintain fish health [130]. The same conclusion, regarding *Lactobacillus* and *Bacillus* genera, can be drawn for the VH10P30 and FH10 diets in this study, while it appears that the modulation of the gut microbial community was slightly more complex in the CV diet. Although the highest microbial diversity was observed in the CV treatment, one of the main constituents of fish intestinal microbiota, the phylum Bacteroidetes, was absent only in this group. Proteobacteria, Bacteroidetes, and Firmicutes are phyla accounting for up to 90% of fish intestinal microbiota in studies to date [131]. The Bacteroidetes are thought to include many symbiotic species that are highly adapted to the human gastrointestinal tract and carry out essential metabolic conversions, often related to the degradation of proteins or complex sugar polymers [132]. This phylum was one of the main components in previous studies of the gut microbiota of European seabass regardless of the introduced diet alterations [38]. Finally, the low activity of IAP in the CV group could have contributed to the absence of the phylum Bacteroidetes since IAP plays an essential role in preserving mucosal tolerance to the resident intestinal microbiome by preventing inflammatory responses [114].

### Effect of experimental diets on seabass skin coloration

Skin coloration plays an important role in consumer acceptance of aquaculture foods, with intense skin coloration usually associated with high-quality products [133]. Nevertheless, skin pigmentation is influenced by a variety of genetic, environmental, physiological, and also dietary factors [134]. The present study has shown that differences in diet composition among fish groups affected skin pigmentation of seabass under similar environmental conditions. Due to the high content of FM, fish fed the CF and FH10 diets expectedly exhibited skin pigmentation most similar to that of farmed or even wild seabass. It should be noted that the differences in external appearance between wild and farmed seabass are mainly due to the intensity of colour expression, with contrasting shades of grey being more pronounced in wild fish, while darker grey colouration is more a common feature observed in farmed seabass [135, 136]. On the other hand, fish fed the CV formulation exhibited the highest percentage of body coverage with the darkest silver-grey hues spreading from the head region to the upper body and fins. However, supplementation of a plant-based diet with PBM and *H. illucens* seemed to have a positive effect on skin coloration. Namely, the VH10P30 and VH10 groups exhibited extensive ventral coverage with lighter hues contrasting with the darker hues on the dorsal side of the body (Fig. 1), thereby having an overall expression of skin pigmentation similar to that of fish fed FM-based diets (CF and FH10). This could be the result of the combination of available pigments in the VH10 and VH10P30 diets. In addition to known sources of natural pigments such as cereal gluten, these two diets also contained *H. illucens* pupae having a carotenoid content ranging from 2.00–2.15 mg/kg depending on the rearing substrate [137, 138] and/or PBM with an estimated carotenoid content of 1.83 mg/kg [139]. A significant improvement in skin colouration has already been demonstrated in gilthead seabream fed diets deprived of fishmeal but enriched with novel protein sources, particularly a mixture of dried marine microalgae and red swamp crab meal [139].

## Conclusions

In conclusion, our results demonstrated that plant-based diets supplemented with pupae meal of *H. illucens* solely or in combination with poultry by-product meal have great potential as an alternative and more sustainable feed for European seabass, without compromising growth performance, intestinal histomorphology, homeostasis, or overall fitness, while maintaining the nutritional value of fillets for human consumption, and even presenting certain beneficial effects. According to the estimated production costs and economic conversion ratios, the VH10 and VH10P30 diets were the most economically sustainable options. Finally, this study also demonstrated the importance of animal proteins in the diet for seabass, as the addition of a small amount of *H. illucens* meal and PBM significantly improved all parameters measured in the VH10 and VH10P30 groups compared to the CV group. It should be noted that the present experimental design included a limited number of replicates per treatment, and so further studies with a more robust design are needed. Since animal proteins are irreplaceable for strictly carnivorous fish species, research on optimal alternatives should be supported to advance the development of environmentally and economically sustainable aquaculture.

## Supplementary Information

**Additional file 1: Supplementary methods.** Description of the environmental and economic sustainability feed indices used in the study, including the Fish-In-Fish-Out Ratio (FIFO) and the Relative Economic Conversion Ratio (rECR). Details of skin colour analysis using the R packages patternize and colordistance for the spatial distribution of colours, the colorDistance function for quantifying colour similarity, and the plotHeat function for visualising the extracted colour patterns along the outline of the seabass body shape. The list of morphometric parameters, including the number of goblet and rodlet cells used for histomorphological evaluation of the seabass intestine fed different test diets. **Table S1.** Protein and lipid content (% as fed), essential amino acid composition (g/kg as fed) of the ingredients used to formulate the test diets for European seabass, and the estimated FIFO and rECR ratios for each diet. **Table S2.** Intestinal morphological measurements of European seabass fed the test diets for 147 d. **Table S3.** Univariate PERMANOVA of alpha diversity based on Euclidean distance for intestinal microbiota of experimentally fed European seabass. **Table S4.** Univariate PERMANOVA of beta diversity based on Bray-Curtis distance for the intestinal microbiota of experimentally fed European seabass. **Fig. S1.** (a) Seawater temperatures and oxygen concentrations in the experimental tanks during the seabass feeding trial (b) Average body weight and growth of fish during the 147-d feeding trial. **Fig. S2.** Distribution of colour distance scores (CDS) according to treatment. **Fig. S3.** Constrained redundancy analysis (RDA) of extracted colour patterns according to feeding treatment. **Fig. S4.** Histological sections of distal intestine of fish fed different test diets. **Fig. S5.** Bar graph of the relative abundance of the European seabass microbiome at the taxonomic family level, according to feeding treatment.

## Data Availability

All data supporting our findings are included in the manuscript.

## Abbreviations

APN: Aminopeptidase N
BBM: Brush border membrane
CDS: Colour distance scores
cDNA: Complementary deoxyribonucleic acid
DI: Distal intestine
DHA: Docosahexaenoic acid
ECR: Economic conversion ratio
EGC: Eosinophil granule cells
EPA: Eicosapentaenoic acid
FAO: Food and Agriculture Organization
FCR: Feed conversion ratio
FI: Feed intake
FIFO: Fish-In-Fish-Out Ratio
IAP: Intestinal alkaline phosphatase
K: Condition factor
L-ANP: Leucine aminopeptidase
LPS: Lipopolysaccharide
Malt: Maltase
MUFA: Monosaturated fatty acids
mRNA: Messenger ribonucleic acid
Na^+^/K^+^ATPase: Sodium/potassium-transporting adenosine triphosphatase
PAPs: Processed animal proteins
PBM: Poultry by-product meal
PC: Pyloric caeca
PepT-1: Peptide transporter 1
PER: Protein efficiency ratio
PI: Proximal intestine
PUFA: Polyunsaturated fatty acids
RGB: Red, Green and Blue
SGR: Specific growth rate
SI: Sucrase-isomaltase
WG: Weight gain
WHO: World Health Organization
