# Supplementary material for: A plant-based diet supplemented with Hermetia illucens alone or in combination with poultry by-product meal: one step closer to sustainable aquafeeds for European seabass
Source: J Anim Sci Biotechnol. 2022 Jul 11;13:77. doi: 10.1186/s40104-022-00725-z (PMC9272557; doi:10.1186/s40104-022-00725-z)
Supplement: Supplementary file 1 — Additional file 1: Supplementary methods. Description of the environmental and economic sustainability feed indices used in the study, including the Fish-In-Fish-Out Ratio (FIFO) and the Relative Economic Conversion Ratio (rECR). Details of skin colour analysis using the R packages patternize and colordistance for the spatial distribution of colours, the colorDistance function for quantifying colour similarity, and the plotHeat function for visualising the extracted colour patterns along the outline of the seabass body shape. The list of morphometric parameters, including the number of goblet and rodlet cells used for histomorphological evaluation of the seabass intestine fed different test diets. Table S1. Protein and lipid content (% as fed), essential amino acid composition (g/kg as fed) of the ingredients used to formulate the test diets for European seabass, and the estimated FIFO and rECR ratios for each diet. Table S2. Intestinal morphological measurements of European seabass fed the test diets for 147 d. Table S3. Univariate PERMANOVA of alpha diversity based on Euclidean distance for intestinal microbiota of experimentally fed European seabass. Table S4. Univariate PERMANOVA of beta diversity based on Bray-Curtis distance for the intestinal microbiota of experimentally fed European seabass. Fig. S1. (a) Seawater temperatures and oxygen concentrations in the experimental tanks during the seabass feeding trial (b) Average body weight and growth of fish during the 147-d feeding trial. Fig. S2. Distribution of colour distance scores (CDS) according to treatment. Fig. S3. Constrained redundancy analysis (RDA) of extracted colour patterns according to feeding treatment. Fig. S4. Histological sections of distal intestine of fish fed different test diets. Fig. S5. Bar graph of the relative abundance of the European seabass microbiome at the taxonomic family level, according to feeding treatment. [file 40104_2022_725_MOESM1_ESM.docx]

**Addition file 1**

**Supplementary methods**

Environmental and economical sustainability feed indices: Fish-In-Fish-Out-ratio (FIFO) which measures the amount of marine ingredients used in the feed, was calculated as follows: FIFO = FCR × (%fishmeal and soluble inclusion + %squid meal inclusion + %fish oil inclusion)/ marine resources yield (22.5%) + fish oil yield (5%)), where FCR is the feed conversion ratio for each feed (<https://www.iffo.com/fifo-data>).

Relative Economic Conversion Ratio (rECR), which measures the cost-effectiveness of fish feed, is expressed in relative terms using feed FH10 as a reference, i.e., the feed with the highest price and is calculated as follows: rECR = (Cost of diet (€/kg) × FCR)/ (Cost of FH10 (€/kg) × FCR FH10) ×100, where FCR is the feed conversion ratio for each feed.

Skin colour analysis: Comparison of colours and their patterns was performed using the patternize and colordistance R packages and by applying the K-means clustering method to extract the dominant colour palette along with its spatial distribution. Colour similarity among treatments was quantitatively measured using the colorDistance function, which uses the earth movers distance metric to compare pixel colour clusters. Normality and homogeneity of variance in the dataset were tested, followed by the Welch ANOVA test of the pairwise colour distance values (CDS) among treatments, along with the Games – Howell post hoc test for pairwise comparisons. Visualisation of the extracted colour patterns along the outline of seabass body shape was performed using the plotHeat function in the patternize R package. For each cluster, the relative proportion of the colour pattern area was calculated in relation to the relative proportion of the total body area.

Intestinal histomorphology: ImageJ was used to determine the following morphometric parameters: the number of goblet cells (total, PAS+, AB+ or AB/PAS+) in six randomly selected fields (×20) and the number of rodlet cells in six randomly selected fields (×40) in the proximal intestine; villus height, villus width, submucosa width, muscularis externa width (×10), lamina propria width (×20) and the number of goblet cells (×20) in six randomly selected fields in the distal intestine. Goblet cells were categorised as PAS+ and AB+ or AB/PAS+, respectively, as the latter population was stained with different shades of dark blue/purple and varied between sections obtained from different other fish. Villus height and width were measured only from intact, unfused villi, and lamina propria width was measured at the centre of these villi.

**Supplementary tables and figures**

**Table S1.** Protein and lipid content (% as fed), essential amino acid composition (g/kg as fed) of ingredient used to formulate the test diets for European seabass subadults, along with estimated fish-in-fish-out ratio (FIFO) and relative economic conversion ratio (ECR) for each diet. The ECR is expressed in relative terms using feed FH10 as a reference, i.e., the feed with the highest price.

| **Protein origin, %** | **CV** | **VH10** | **VH10P30** | **FH10** | **CF** |
| --- | --- | --- | --- | --- | --- |
| Fish | 15 | 15 | 15 | 85 | 85 |
| PBM | - | - | 30 | - | - |
| *Hermentia illucens* | - | 10 | 10 | - | 10 |
| Protein from veg mix + AA | 85 | 75 | 45 | 15 | 5 |
| **Amino acid (AA) composition, g/kg** |  |  |  |  |  |
| Essential AA composition |  |  |  |  |  |
| Arginine | 27.4 | 26.9 | 29.4 | 24.0 | 24.7 |
| Histidine | 10.7 | 10.7 | 10.1 | 11.3 | 11.2 |
| Isoleucine | 18.5 | 17.7 | 15.5 | 16.1 | 16.7 |
| Leucine | 38.0 | 36.4 | 31.8 | 28.8 | 29.2 |
| Lysine | 25.2 | 25.5 | 23.3 | 29.3 | 29.4 |
| Methionine + cysteine | 18.1 | 17.8 | 16.8 | 13.0 | 13.0 |
| Phenylalanine +tyrosine | 35.7 | 35.5 | 31.4 | 35.3 | 34.8 |
| Threonine | 16.2 | 16.3 | 17.1 | 16.6 | 16.4 |
| Tryptophan | 4.4 | 4.4 | 4.5 | 4.8 | 4.7 |
| Valine | 20.4 | 20.9 | 20.8 | 20.3 | 19.6 |
| Non-essential AA composition |  |  |  |  |  |
| Aspartic acid | 34.5 | 35.9 | 39.4 | 35.7 | 34.0 |
| Glutamic acid | 91.3 | 84.6 | 75.5 | 49.8 | 53.5 |
| Alanine | 20.8 | 21.9 | 25.6 | 26.2 | 24.3 |
| Glycine | 20.0 | 20.6 | 28.8 | 28.7 | 27.8 |
| Proline | 31.7 | 30.6 | 29.6 | 20.3 | 20.1 |
| Serine | 21.1 | 20.8 | 19.8 | 16.6 | 16.4 |
| **Lipid origin, %** |  |  |  |  |  |
| Fish | 34 | 34 | 34 | 66 | 66 |
| Lipid from alternate ingredients | 66 | 66 | 66 | 34 | 34 |
| **Sustainability indices** |  |  |  |  |  |
| FIFO | 1.00 | 0.69 | 0.75 | 4.06 | 3.88 |
| rECR | 89.3 | 68.9 | 60.1 | 100.0 | 87.8 |

**Table S2.** Intestinal morphological measurements of European seabass fed with test diets over 147 d.

| **Parameter** | **CV** | **VH10** | **VH10P30** | **FH10** | **CF** | ***P* value** |
| --- | --- | --- | --- | --- | --- | --- |
| **Proximal intestine** | | | | | | |
| **GC** | 72.56±2.12^ab^ | 64.51±2.15^c^ | 87.46±2.20^c^ | 78.86±2.00^a^ | 68.83±2.49^bc^ | 0.001 |
| **AB or AB/PAS** | 53.52±1.94^a^  (AB/PAS) | 39.99±1.62  (AB) | 70.81±1.74  (AB) | 64.04±1.80  (AB) | 51.64±1.85^a^  (AB/PAS) | 0.001 |
| **PAS** | 19.05±0.92^a^ | 14.88±1.11^bc^ | 20.18±1.19^a^ | 13.11±0.73^bc^ | 15.36±1.13^c^ | 0.001 |
| **RC** | 11.11±0.51^a^ | 11.11±0.49^a^ | 18.86±0.92^b^ | 19.00±0.78^b^ | 13.64±0.59 | 0.001 |
| **Distal intestine** | | | | | | |
| **V_H_** | 372.19±9.54^ab^ | 305.11±7.02 | 393.70±8.72^ac^ | 344.88±7.76^b^ | 398.55±5.64^c^ | 0.001 |
| **V_W_** | 89.68±1.63^acd^ | 83.12±1.33^b^ | 86.59±1.62^abcd^ | 88.96±1.43^cd^ | 89.93±1.54^d^ | 0.006 |
| **LP** | 15.63±0.43^ad^ | 18.41±0.80^bc^ | 17.14±0.44^bd^ | 16.59±0.56^abcd^ | 16.16±0.46^d^ | 0.017 |
| **SM** | 59.93±2.02^ab^ | 46.77±1.32 | 63.84±1.47 | 57.53±1.19^ac^ | 61.08±1.43^bc^ | 0.001 |
| **ME** | 134.87±2.94^a^ | 124.13±1.98 | 143.18±2.82^a^ | 159.57±3.45^b^ | 155.79±3.05^b^ | 0.001 |
| **GC** | 198.53±5.37 | 220.33±5.61^a^ | 218.43±4.41^a^ | 229.21±5.71^a^ | 174.61±5.76 | 0.001 |

GC, number of goblet cells; AB or AB/PAS, number of alcian blue or alcian blue/periodic acid-Schiff positive cells (containing acidic or acidic and neutral mucins, respectively); PAS, number of periodic acid-Schiff positive cells (containing neutral mucins); RC, number of rodlet cells; VH, villus height; VW, villus width; LP, lamina propria width; SM, submucosa width; ME, muscularis externa width. Data in brackets for AB or AB/PAS indicate the predominance of either of the two cell types. Values are represented as mean ± standard error of the mean (SEM). Row means indicated with different superscript letter differ significantly (Tukey test; *P* < 0.05).

**Table S3.** Summary of one-way univariate PERMANOVA of alpha diversity based on Euclidean distance, *P*-values obtained after 999 unrestricted permutations of raw data with Monte-Carlo simulation included. PERMANOVA tested for differences in observed richness of intestinal microbiota of experimentally fed European seabass between feeding treatments (five levels: CV, VH10, VH10P30, CF, FH10). Full description of each feeding treatment is available in Material and Methods.

|  | **Source of variation** | **df** | **MS** | **F** |
| --- | --- | --- | --- | --- |
| Main test | Feeding treatment | 4 | 2584.9 | 4.93^*^ |
|  | Residual | 13 | 524.55 |  |
|  |  |  |  |  |
| Pair wise tests | Significant interaction between CV and VH10 treatment^**^; CV and CF treatment^**^; and VH10 and CF treatment^*^ | | | |

^*^Statistically significant difference at *P* < 0.05; ^**^Statistically significant difference at *P* < 0.01;

df – degrees of freedom; MS – mean sum of squares; F – F value.

**Table S4.** Summary of one-way univariate PERMANOVA of beta diversity based on Bray Curtis distance, *P*-values obtained after 999 unrestricted permutations of log transformed data with Monte-Carlo simulation included. PERMANOVA tested for differences in beta diversity of intestinal microbiota of experimentally fed European seabass between feeding treatments (five levels: CV, VH10, VH10P30, CF, FH10). Full description of each feeding treatment is available in Material and Methods.

|  | **Source of variation** | **df** | **MS** | **F** |
| --- | --- | --- | --- | --- |
| Main test | Feeding treatment | 4 | 3005.6 | 1.6377^*^ |
|  | Residual | 13 | 1835.3 |  |
|  |  |  |  |  |
| Pair wise tests | Significant interaction between CV and VH10 treatment^*^; CV and CF treatment^*^ | | | |

^*^Statistically significant difference at *P* < 0.05; df – degrees of freedom; MS – mean sum of squares; F – F value.

| 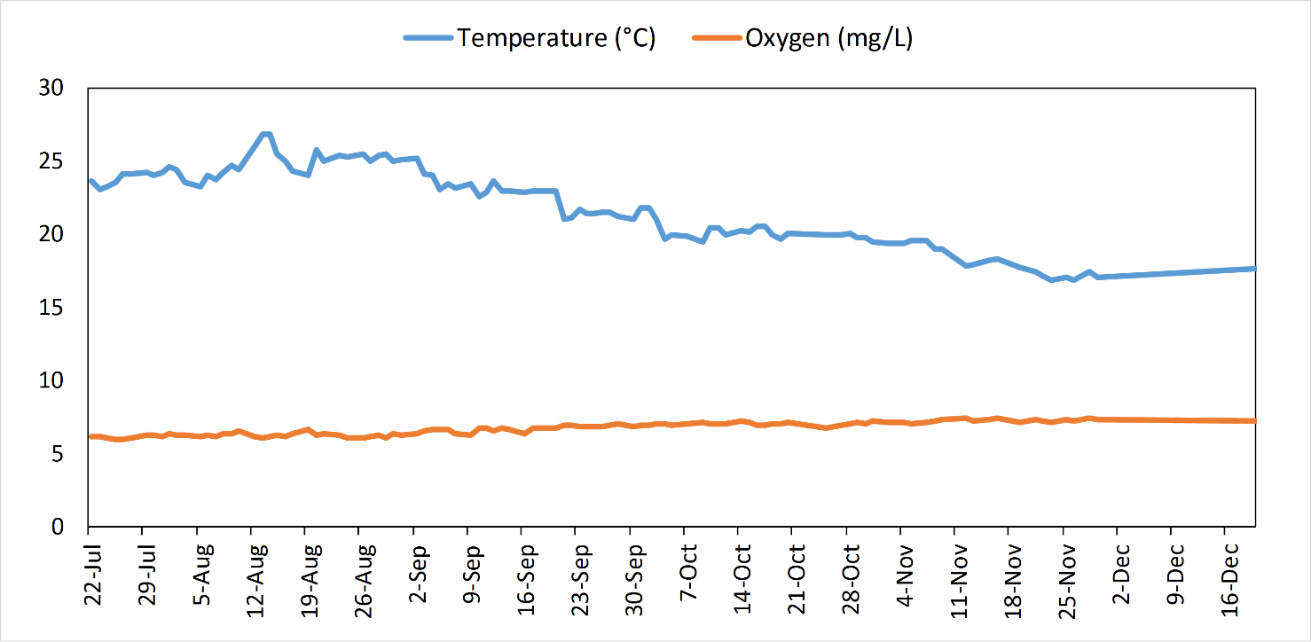  a) |
| --- |
| 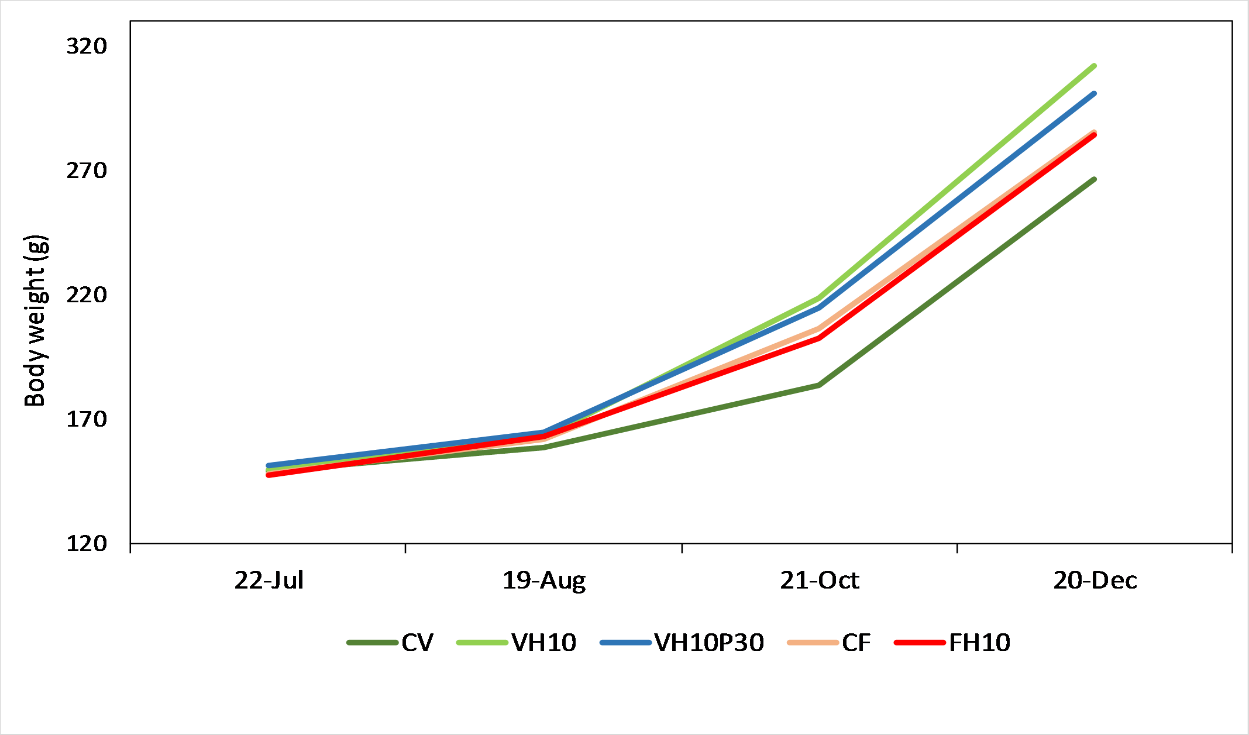  b) |
| **Fig. S1**. **a)** Seawater temperatures and oxygen concentrations in the experimental tanks during the seabass feeding trial in 2019. In addition, each tank used in the trial was supplied with 15 L/min of flow-through, mechanically pre-filtered marine water, obtained from an adjacent coastal pump station and aerated with air pumps. The natural photoperiod followed seasonal changes (July–December) and ranged from 14.42 to 9.12 h of daylight. Water temperature ranged from 24.5 ± 0.9 °C during July-August to 17.2 ± 1.4 °C during November-December, dissolved oxygen content ranged from 6.0 to 7.5 mg/L, and salinity ranged from 37.2 to 38‰. **b**) Average body weight and growth of fish during the 147-d feeding trial. Feeding trials were conducted in two replicate tanks for each experimental diet (CV, VH10, VH10P30, CF, FH10). Body weight was calculated for each diet as the mean across replicate tanks (error bars were omitted for simplicity). |

| 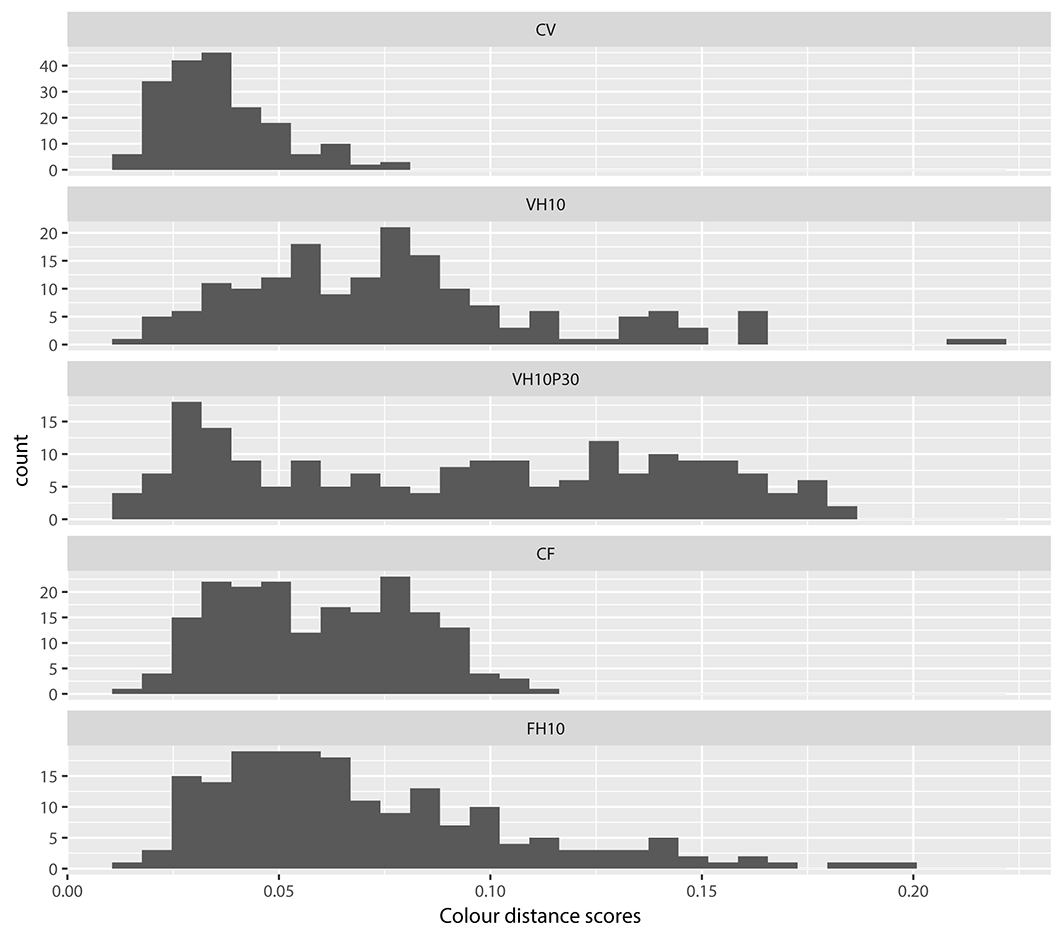 |
| --- |
| **Fig. S2.** Distribution of colour distance scores (CDS) according to treatment. |

| 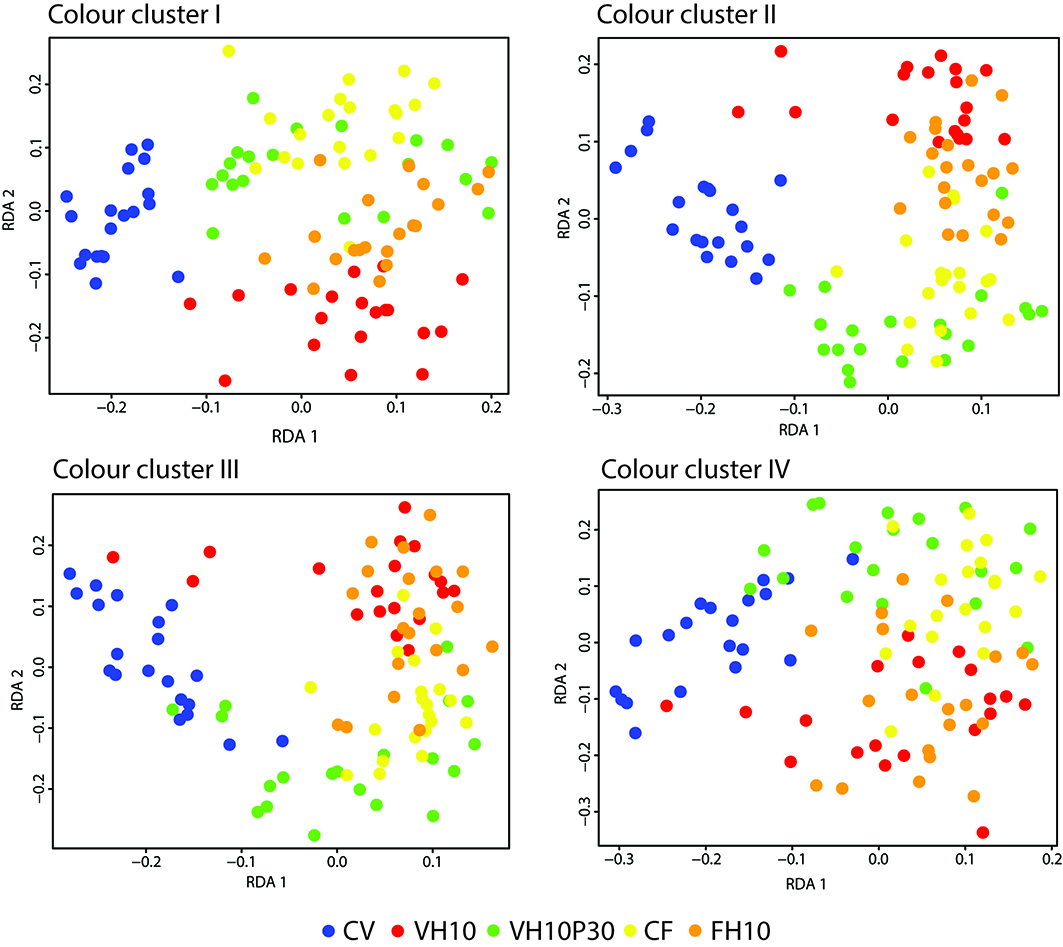 |
| --- |
| **Fig. S3.** Constrained redundancy analysis (RDA) of extracted colour patterns per treatment (blue – CV; red – VH10; green –VH10P30; yellow – CF; orange –FH10). |

| 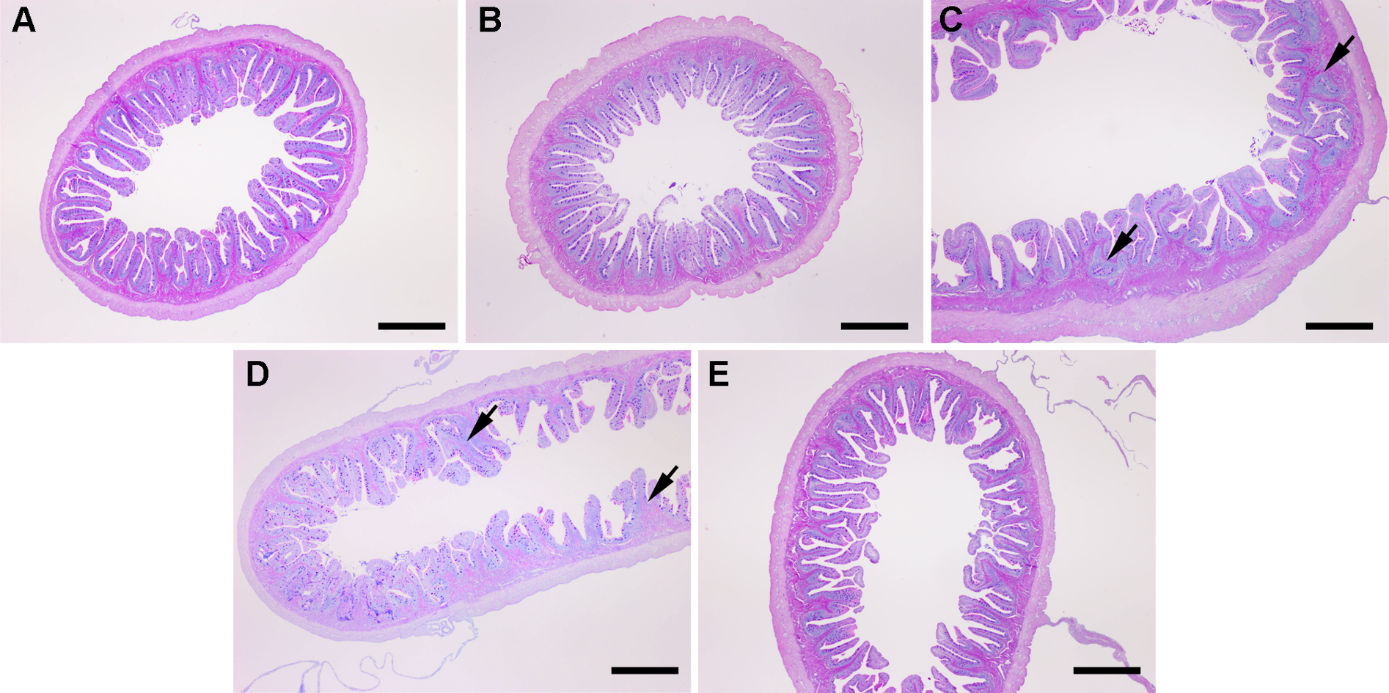 |
| --- |
| **Fig. S4.** Distal intestine histological sections of fish fed different test diets. **A** CF diet, well preserved intestinal morphology with elongated and regular *villi*. **B** FH10 diet, similar as with CF diet intestinal morphology was well preserved with elongated and regular *villi*. **C** CV diet, irregularly shaped *villi* with extensive multifocal lateral fusion (arrows). **D** VH10 diet, irregularly shape *villi* with multifocal lateral fusion (arrows). **E** VH10P30 diet, irregularly shaped *villi* with focal minor lateral fusion. Alcian blue/PAS staining, pH=2.5. Scale bar=500 µm. |

| 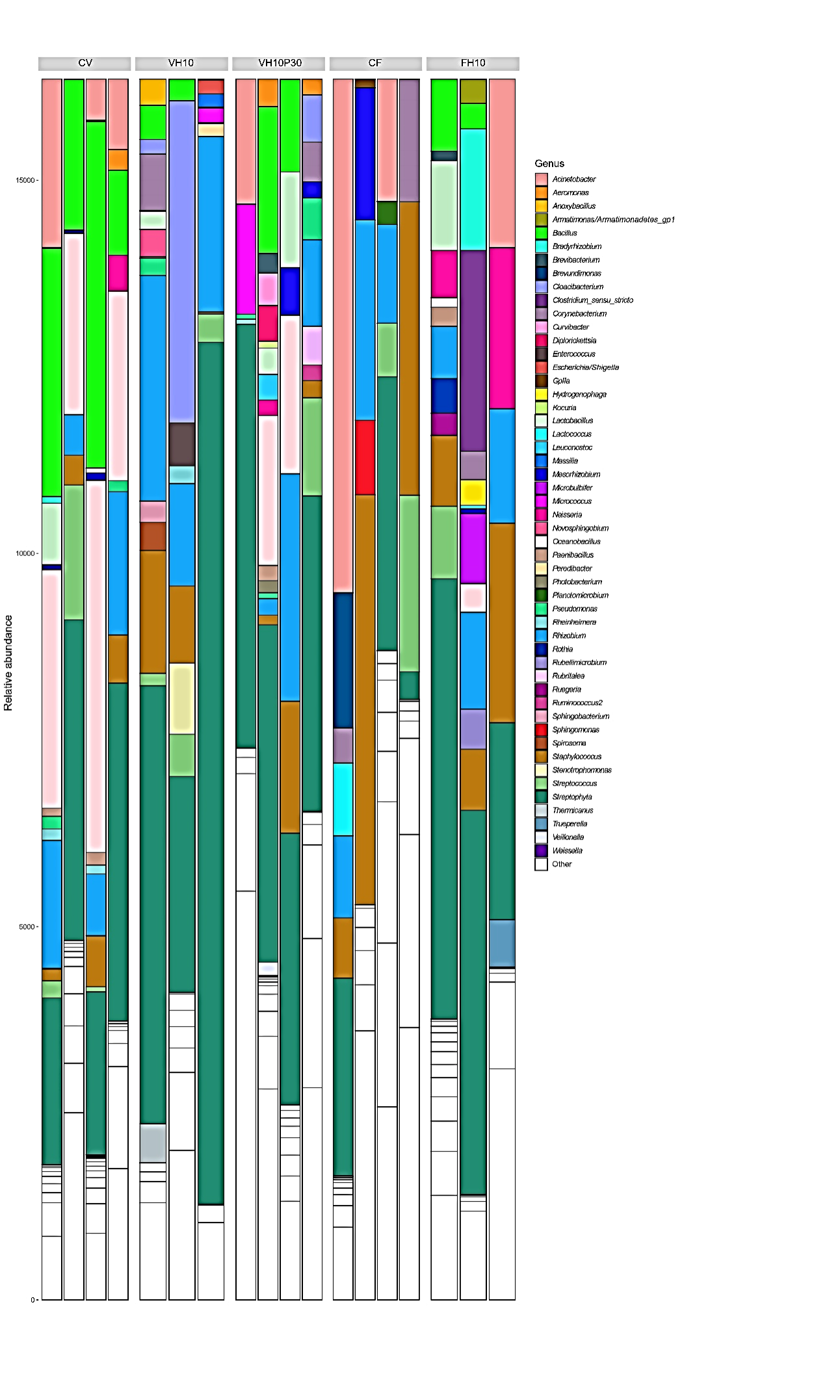 |
| --- |
| **Fig. S5.** Subadult European seabass microbiome relative abundance barplot at family taxonomic level, according to feeding treatment. Color-coded genera legend is given next to the barplot. |
